# How bacterial xenogeneic silencer rok distinguishes foreign from self DNA in its resident genome

**DOI:** 10.1093/nar/gky836

**Published:** 2018-09-25

**Authors:** Bo Duan, Pengfei Ding, Timothy R Hughes, William Wiley Navarre, Jun Liu, Bin Xia

**Affiliations:** 1 Beijing Nuclear Magnetic Resonance Center, College of Chemistry and Molecular Engineering, School of Life Sciences, Peking University, Beijing 100871, China; 2 Department of Molecular Genetics, Donnelly Centre for Cellular and Biomolecular Research, University of Toronto, Toronto, Ontario, Canada

## Abstract

Bacterial xenogeneic silencers play important roles in bacterial evolution by recognizing and inhibiting expression from foreign genes acquired through horizontal gene transfer, thereby buffering against potential fitness consequences of their misregulated expression. Here, the detailed DNA binding properties of Rok, a xenogeneic silencer in *Bacillus subtilis*, was studied using protein binding microarray, and the solution structure of its C-terminal DNA binding domain was determined in complex with DNA. The C-terminal domain of Rok adopts a typical winged helix fold, with a novel DNA recognition mechanism different from other winged helix proteins or xenogeneic silencers. Rok binds the DNA minor groove by forming hydrogen bonds to bases through N154, T156 at the N-terminal of α3 helix and R174 of wing W1, assisted by four lysine residues interacting electrostatically with DNA backbone phosphate groups. These structural features endow Rok with preference towards DNA sequences harboring AACTA, TACTA, and flexible multiple TpA steps, while rigid A-tracts are disfavored. Correspondingly, the *Bacillus* genomes containing Rok are rich in A-tracts and show a dramatic underrepresentation of AACTA and TACTA, which are significantly enriched in Rok binding regions. These observations suggest that the xenogeneic silencing protein and its resident genome may have evolved cooperatively.

## INTRODUCTION

Horizontal gene transfer (HGT), which allows bacteria to acquire new genes and phenotypes much faster than the gradual modification of pre-existing genes, plays an important role in bacterial evolution (1–3). Xenogeneic (foreign-derived) genes acquired through HGT have the potential to increase bacterial fitness in response to specific environments (e.g. antibiotic resistance) (4). However, newly acquired genes can often have deleterious effects on fitness when expressed improperly by hindering normal cellular processes, disrupting established regulatory networks, or wasting resources (5). Bacteria use xenogeneic silencers to control the expression of foreign genes to avoid these adverse effects, allowing them to retain pools of potentially useful genes. Bacteria may find proper ways to use these genes during evolution (6).

It is known that for many types of bacteria, xenogeneic genes often have a higher AT-content than their resident genomes (7,8). To date, four families of AT-rich DNA binding proteins have been identified as xenogeneic silencers: the H-NS-like proteins mainly found in alpha-, beta- and gamma-proteobacteria (9), the Lsr2-like proteins from actinobacteria (10), the MvaT-like proteins found in pseudomonads and some other gamma-proteobacteria (11), and more recently, the Rok-like proteins of some Gram-positive bacilli including *B. subtilis* (12). All four families of xenogeneic silencers bind AT-rich DNA sequences via their C-terminal domains, which share almost no sequence or structural homology between families (12–15). The N-terminal domains of H-NS, Lsr2 and MvaT can oligomerize and form nucleoprotein filaments with DNA to inhibit transcription (16–20), and the N-terminal domain of Rok was also proposed to have a similar function (12). For H-NS, Lsr2 and MvaT, the structures of their C-terminal DNA binding domains and the mechanisms for AT-rich DNA sequence recognition have been elucidated (13–15), but the specific DNA binding mechanism of Rok remains unknown.

Rok was originally identified as the repressor of *comK*, the master regulator of competence pathway in *Bacillus* (21). Rok binds directly to the promoter region of *comK* and represses its transcription. Both Rok and ComK can bind to the *rok* promoter and repress *rok* transcription, forming a feedback loop (21). Further studies show that several gene clusters are also negatively regulated by the direct binding of Rok, including many genes involved in cell surface and extracellular functions such as the genes related to antibiotic production (22,23). Under certain circumstances, the activity of Rok is modulated by the bacterial replication initiator and transcription factor DnaA. Thirty six genome regions were found to be associated with both Rok and DnaA. The presence of DnaA enhances the repression function of Rok, while DnaA itself is unable to repress these genes (24–26). The genome-wide binding profile of Rok obtained by ChIP-chip and ChIP-seq experiments show that the chromosomal regions bound by Rok display relatively high AT-content, which is suggestive of horizontal transfer (12,24). These findings indicate that Rok may function as a xenogeneic silencer in *Bacillus*.

In *Bacillus subtilis*, the full-length Rok protein contains 191 amino acids and has three distinct regions based on sequence conservation analysis. Region I (residues 1–45) and region III (residues 96–191) are highly conserved, while region II (residues 46–95) is less conserved. Region III was predicted to belong to the winged helix protein family with a DNA/RNA-binding 3-helical bundle and is necessary and sufficient for DNA recognition, while regions I and II were suggested to contribute to DNA binding through oligomerization (12). Rok was proposed to be a major groove binding protein as the major groove binding ligand methyl green prevented the shifting of DNA by Rok in EMSA experiments, while the minor groove binding drug chromomycin A3 did not (27).

In this work, the DNA sequence binding preferences of Rok were characterized using protein binding microarrays (PBM) (28), and solution structures of its C-terminal DNA binding domain were determined both free and in complex with DNA. The results demonstrate that the structure and the DNA recognition mechanism of Rok are distinct from other xenogeneic silencers. Notably, Rok displays a degree of sequence specificity not observed with H-NS, Lsr2 or MvaT. Sequence motifs preferred by Rok are conspicuously depleted in its resident genome, which helps Rok distinguish xenogeneic genes efficiently.

## MATERIALS AND METHODS

### Protein binding microarray (PBM)

GST-tagged Rok and Rok-C^97-191^ proteins were expressed using T7-GST vector pTH5325 (pDEST15-Magic_modified) and purified with GST affinity column and size exclusion chromatography. Then the proteins were applied to microarrays containing 41 944 double-stranded 60-mer oligonucleotide target sequences. Each sequence is composed of a 25-mer constant primer sequence and a 35-mer variable sequence generated by partitioning a de Bruijin sequence of order 10. Every non-palindromic 8-mer is represented at least 32 times (16 times for palindromic 8-mers) on each array. The binding preferences of Rok and Rok-C^97-191^ for all 8-mer DNA sequences were quantified using the enrichment score (E-score), which is rank-based, unitless, and has a nonlinear scaling with signal intensity, ranging from 0.5 (most favored) to −0.5 (most disfavored). Among all 32 896 different 8-mer DNA sequences, 157 and 132 8-mers achieved an E-score above 0.40 for Rok and Rok-C^97-191^, respectively. 8-mers with *E*-score above 0.40 were used for MEME motif searches.

### NMR sample preparation

The coding sequence of Rok was amplified by PCR from the *B. subtilis* subsp. subtilis str. 168 genome, and DNA fragments encoding Rok, Rok-N^1-95^, Rok-C^97-191^, Rok-C^102-185^, and Rok-C^114-185^ were cloned into the NdeI and XhoI sites of pET-21a (+) expression vector (Novagen), followed by a C-terminal His_6_-tag. Point mutations of Rok-C^114-185^ were generated using the site-directed mutagenesis kit (SBS Genetech). The plasmids were then transfected to *Escherichia coli* Rosetta (DE3) competent cells. Bacteria were cultured in Luria-Bertani (LB) medium at 35°C and protein expression was induced with 0.1 mM IPTG at OD_600_ 0.8 for 4 h. For the preparation of NMR samples, ^15^N and ^13^C labeled M9 minimal medium were used instead of the LB medium. The cells were harvested by centrifugation and resuspended in lysis buffer (50 mM sodium phosphate, 1 M NaCl, 20 mM imidazole, pH 8.0). After sonication and centrifugation, the supernatant was applied to a Ni-NTA affinity column (Qiagen). The column was then washed with the lysis buffer, and His_6_-tagged proteins were eluted with elution buffer (50 mM sodium phosphate, 1 M NaCl, 250 mM imidazole, pH 8.0). For Rok-C^114-185^ mutants with a Lys to Ala or Arg to Ala mutation (p*I* ≈ 8.0), the pH value of the lysis and elution buffer was adjusted to 9.0. Proteins were further purified using size exclusion chromatography with a Superdex 75 Gel Filtration Column (GE), in 50 mM sodium phosphate buffer with 50 mM NaCl (pH 6.0).

For the DNA targets used in our binding and structural assays, synthesized DNA oligos were dissolved in 50 mM sodium phosphate buffer with 50 mM NaCl (pH 6.0), heated to 94°C for 5 min and then annealed by slowly cooling down to room temperature.

### NMR data collection

All NMR samples were in 50 mM sodium phosphate, 50 mM NaCl (pH 6.0) with 90% H_2_O/10% D_2_O, along with 0.01% NaN_3_ and 0.01% DSS.

The NMR sample for the structure determination of free Rok-C^97–191^ contained 1 mM uniformly ^15^N,^13^C-labeled protein. The following experiments were performed at 298 K for resonance assignments and structure determination: 2D ^1^H–^15^N HSQC, 2D ^1^H–^13^C HSQC, 3D HNCA, 3D HN(CO)CA, 3D HNCACB, 3D CBCA(CO)NH, 3D HNCO, 3D HBHA(CBCA)(CO)NH, 3D (H)CCH-COSY, 3D (H)CCH-TOCSY, 3D H(C)CH-TOCSY, 3D H(C)CH-COSY, 3D ^1^H–^15^N-edited NOESY-HSQC, 3D ^1^H–^13^C-edited NOESY-HSQC and 3D ^1^H–^13^C-edited-NOESY optimized for aromatic resonances (29).

For the structure determination of the Rok-C^102–185^/Seq1 complex, the ratio of the two molecules and the experiment temperature were optimized according to the 2D ^1^H–^15^N HSQC and 2D F1, F2-^15^N/^13^C-filtered ^1^H-^1^H NOESY spectra. The optimized NMR sample contained 0.5 mM uniformly ^15^N, ^13^C-labeled Rok-C^102–185^ and 2.5 mM unlabeled Seq1 DNA, and all NMR spectra were collected at 308 K. In addition to the NMR experiments mentioned above, 2D F1, F2-^15^N/^13^C-filtered ^1^H–^1^H NOESY experiments were used for the resonance assignments and structure determination of Seq1 DNA, and 3D F1-^15^N/^13^C filtered, F2-^13^C edited NOESY-HSQC experiments were used to distinguish inter- and intra-molecular NOE signals in the 3D ^1^H–^13^C-edited NOESY-HSQC spectrum (29).

500, 700 or 950 MHz Bruker AVANCE spectrometers equipped with triple-resonance cryoprobes were used for data collection. The Bruker standard pulse sequences were used for all NMR experiments. Proton chemical shifts were referenced directly to internal DSS. ^15^N and ^13^C chemical shifts were referenced indirectly to DSS.

### Solution structure determination

For the structure calculation of free Rok^97–191^, distance restraints derived from 3D ^1^H–^15^N-edited NOESY-HSQC and 3D ^1^H–^13^C-edited NOESY-HSQC experiments and protein dihedral angle restraints obtained using TALOS+ were used (30). Initial structures generated by the CANDID module of CYANA were used as filter models to refine the NOE assignments and distance restraints using SANE (31,32). Then the refined restraints were used for the next round structure calculation using the DYANA module, generating new models for SANE. This procedure was carried out iteratively until no distance violation larger than 0.5 Å existed. Then 200 structures were calculated by CYANA, and the 100 structures with the lowest target function values were selected for further refinement using AMBER 12 and SANE iteratively.

For the structure calculation of the Rok-C^102–185^/Seq1 complex, structures of Rok-C^102–185^ and Seq1 DNA were calculated separately at first. The structure calculation procedures of Rok-C^102–185^ were similar to that of free Rok-C^97–191^. The structure of Seq1 DNA was calculated using AMBER 12 with distance restraints derived from 2D F1, F2-^15^N/^13^C-filtered NOESY spectra and empirical torsion angle restrains and Waston–Crick restrains of B-form DNA according to the AMBER tutorial. The coordinates of protein and DNA structures were then arbitrarily combined in 100 different initial states. Intermolecular NOEs obtained through analyzing the 3D F1-^15^N/^13^C filtered, F2-^13^C edited NOESY-HSQC and 3D ^1^H–^13^C-edited NOESY-HSQC spectra were gradually added for complex structure calculation using AMBER 12 and SANE iteratively. During this process, the intermolecular distance restraints were initially set to 50 Å, and gradually decreased to 20, 16, 12 and 8 Å, and then to proper restraint distances based on signal volumes, while the force constant was gradually increased from 0 to 2, 10, and finally to 20 kcal/mol·Å.

The structure refinement was completed when over 95% of the NOE restraints were properly used and there was no angle violation bigger than 5° and no distance violation bigger than 0.2 Å. Finally, the top 20 structures with the lowest AMBER energy were selected and an energy minimized mean structure was generated. The member of the ensemble that is closest to the mean structure (conformer 4 of free Rok^97-191^; conformer 11 of Rok^102–185^/DNA complex) was used for structure presentation (33). Protein structure quality was analyzed using PROCHECK-NMR and DNA base-step, groove and helical parameters were analyzed using CURVES+ (34,35).

### Isothermal titration calorimetry (ITC)

ITC experiments were performed using the MicroCal PEAK system (Malvern). Protein or DNA samples were prepared in sodium phosphate buffer with 200 mM NaCl (pH 6) and experiments were performed at 277 K to stabilize the double helix structure of DNA (36). 50 μM protein was placed in the cell and titrated with 750 μM Seq1 DNA. DNA to buffer titration experiments were performed as controls. The data were analyzed using the MicroCal PEAQ-ITC Analysis Software. Each measurement is repeated twice, with one of the curves shown in the supplementary figures.

### Genome analysis

The genomic AT-content of each *Bacillus* species is obtained from NCBI genome database (ftp://ftp.ncbi.nlm.nih.gov/genomes/GENOME_REPORTS/). *Bacillus* species with ≥2 sets of genomic sequence data were analyzed. A Blastp search against the non-redundant database of NCBI with the sequence of Rok-C^114–185^ was used to find Rok homologs. The number of Rok homologs found in a certain species and the number of sequenced genomes for this species were counted, and their ratio was calculated. A species is considered to be Rok-containing if the ratio is >0.5, while a ratio below 0.1 indicates lack of Rok in the species. The occurrence counts of 512 different 5-bp sequences in a certain genome were divided by the length of the genome to yield the occurrence frequencies. Complementary sequences such as AAAAA and TTTTT are regarded as the same one.

The sequences of Rok binding regions mapped by Seid *et al.* (24) using ChIP-seq were extracted from the *B. subtilis* genome and each was extended to 300 bp centered by the reported 40 bp binding peak. The occurrence frequency of each 5-bp sequence in the Rok binding regions was calculated similarly as above, and was then divided by its occurrence frequency in the *B. subtilis* genome to calculate the relatively enrichment fold.

## RESULTS

### Domain architecture of Rok

It has been demonstrated that the C-terminal domain of Rok is responsible for its DNA binding activity (12). However, no experimental data is available for the oligomerization of the N-terminal domain. Thus, we expressed the full-length Rok protein, as well as its N-terminal domain (Rok-N^1–95^, residues 1–95) and C-terminal domain (Rok-C^97–191^, residues 97–191) in *E. coli*, and all proteins were purified solubly. The oligomerization states of Rok, Rok-N^1–95^, and Rok-C^97–191^ were analyzed using gel filtration chromatography with a GE Hiload™ 16/600 Superdex™ 75 preparation grade column. The elution volume of Rok-C^97–191^ (theoretical MW: 11.8 kD) was about 80 ml, which was close to that of cytochrome C (12.4 kD), suggesting that Rok-C^97–191^ exists as a monomer in solution. The elution volumes of both full-length Rok (22.9 kD) and Rok-N^1–95^ (12.4 kD) were near the void volume of the column, suggesting that Rok multimerizes through its N-terminal domain into a higher order oligomer (Supplementary Figure S1A). Therefore, the overall domain architecture of Rok is similar to other xenogeneic silencers H-NS, Lsr2 and MvaT, with an N-terminal oligomerization domain and a monomeric C-terminal DNA binding domain. It is very likely that Rok could also form nucleoprotein filaments when binding DNA, just as H-NS, Lsr2 and MvaT do.

### DNA binding preferences of Rok

**Figure 1.**
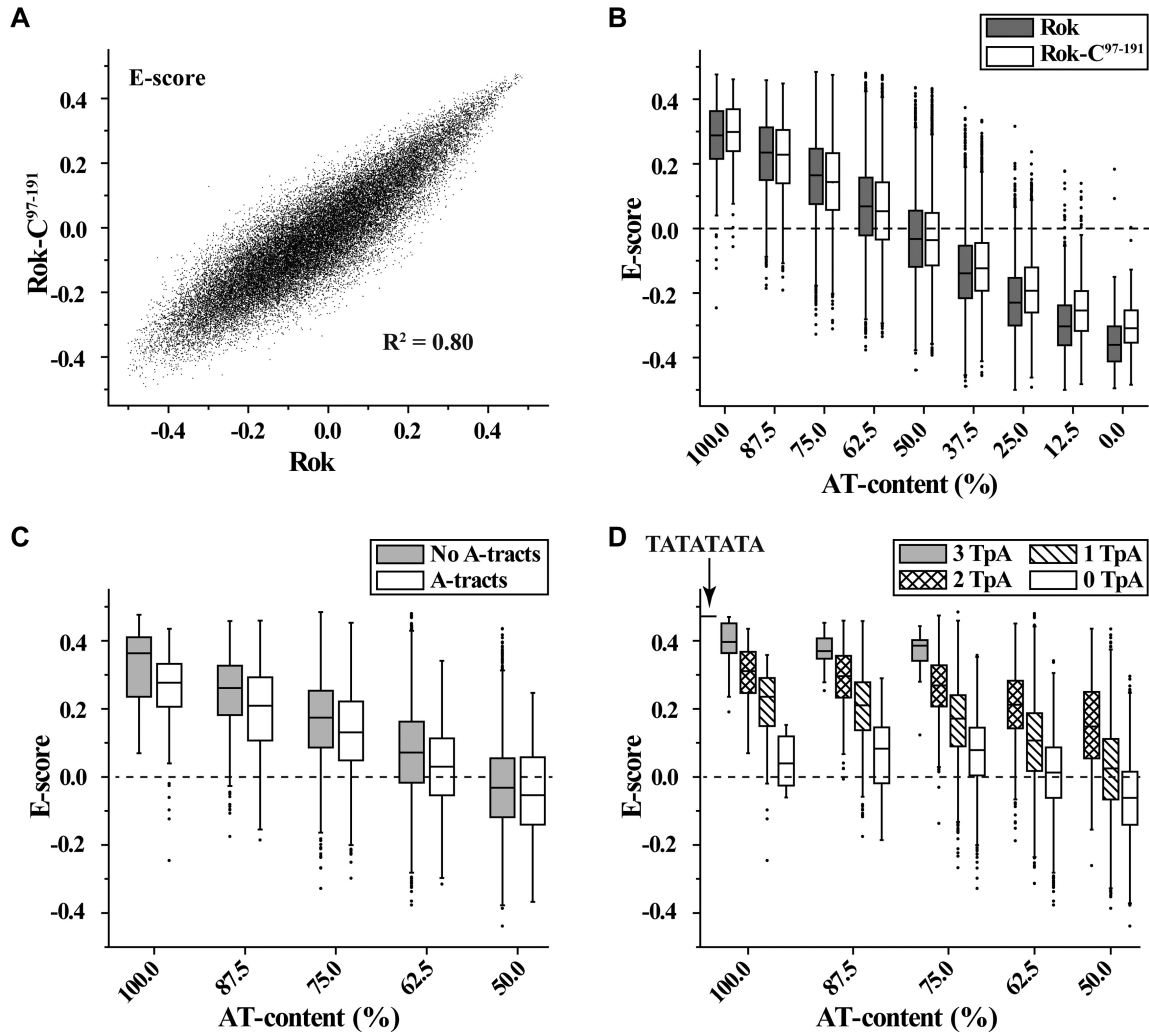
DNA binding preferences of Rok from protein binding microarray analysis. *E*-score reflects the relative affinity for a particular 8-mer sequence. Box plots show the *E*-score distributions of 8-mers with certain characteristics. Bands at the bottom, top, and inside of the box represent the first quartile, the third quartile, and the median, respectively. The whiskers indicate points within the 1.5 interquartile range, and the black dots represent the outliers. (**A**) Scatter plots comparison of the *E*-scores of all individual 8-mers for full length Rok protein and its DNA binding domain Rok-C^97–191^. (**B**) E-score distributions of 8-mers with different AT-contents for Rok and Rok-C^97-191^. (**C** and **D**) The influence of A-tracts and TpA steps on 8-mer *E*-scores for Rok.

The DNA binding preferences for the full-length Rok and its C-terminal domain were characterized using protein binding microarrays (PBM) (28). The E-scores of PBM data are well correlated for all 8-mers between Rok and Rok-C^97–191^ (Figure 1A), and indicate that both of them prefer to bind DNA sequences with high AT-content (Figure 1B). For 8-mers with 4 or more AT base pairs, the existence of A-tracts (A_*n*_T_*m*_, *n* ≥ 0, *m* ≥ 0, *n*+*m* ≥ 4) decreases the binding affinity of Rok, which is most prominent for 8-mers with 100% AT-content (Figure 1C). On the contrary, TpA steps significantly enhance the binding, as 8-mers with more TpA steps give remarkably higher *E*-scores (Figure 1D). For AT-rich DNA sequences, A-tracts form rigid structures with narrow minor grooves, while TpA steps are more flexible and have relatively wider minor grooves (37,38). These findings imply that besides AT-content, the flexibility and the minor groove width of DNA affect the binding of Rok.

**Figure 2.**
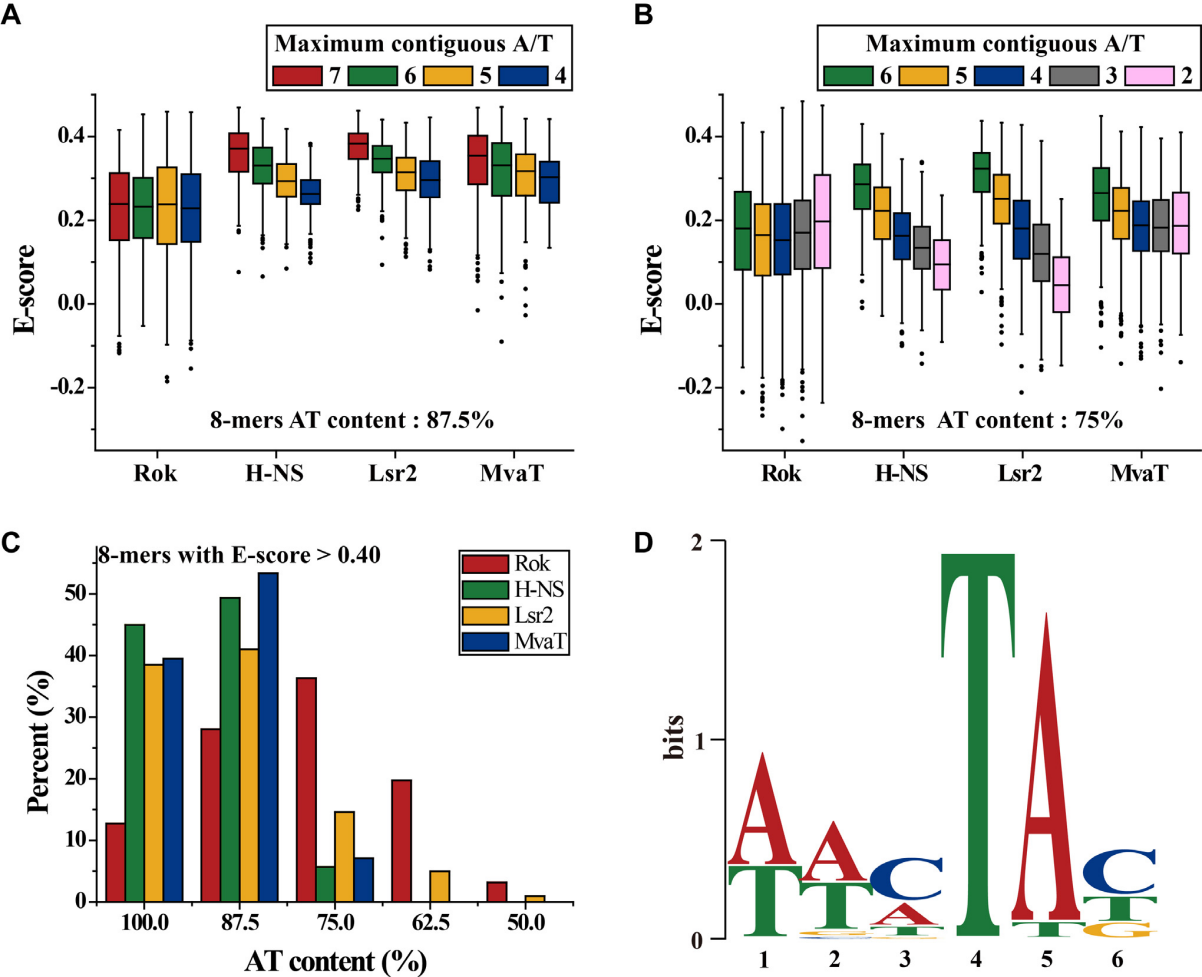
Comparisons of the effect of G/C insertions on AT-rich DNA binding preferences of Rok with other xenogeneic silencers H-NS, Lsr2 and MvaT. The PBM data for H-NS, Lsr2 and MvaT were published previously (13,14). (**A** and **B**) The influence of G/C insertions on DNA binding for Rok, H-NS, Lsr2 and MvaT. (**C**) AT-content distributions of 8-mers with *E*-score above 0.40 for Rok, H-NS, Lsr2 and MvaT. (**D**) MEME motifs generated from 8-mer sequences with *E*-score above 0.40 for Rok.

In contrast to what has been observed for other xenogeneic silencers, Rok binding (*E*-scores) is not affected by the presence of a single GC base-pair in its binding target (Figure 2A). Among the 8-mer motifs with 2 GC base pairs, *E*-scores are highest for those with the general pattern of WWSWWSWW (W, A/T; S, G/C) (Figure 2B). Analysis of all the high affinity 8-mers with *E*-scores >0.40 revealed that most of the sequences contain at least one GC base pair, and there are more 8-mers with 2 GC base pairs than sequences composed entirely of AT base pairs (Figure 2C). A 6-bp MEME motif was discovered using all 8-mer sequences with *E*-scores above 0.40 for Rok (Figure 2D), which contains a conserved TpA step at the fourth and fifth positions. The first two positions of this motif are A or T, while C is relatively more preferred at the third and sixth positions. This motif is representative as it is found in 139 of all the 157 8-mers with *E*-scores >0.40.

The motifs targeted by Rok are quite different from those targeted by H-NS, Lsr2 and MvaT, which uniformly prefer 8-mers with longer contiguous A/T sequences, although MvaT shows some tolerance for G/C interruptions (14,15). High affinity 8-mers (*E*-scores >0.40) for H-NS, Lsr2 and MvaT are mainly sequences with 100% or 87.5% AT-content (Figure 2C), and no representative sequence specific DNA motif could be identified.

### Solution structure of the C-terminal domain of Rok

**Figure 3.**
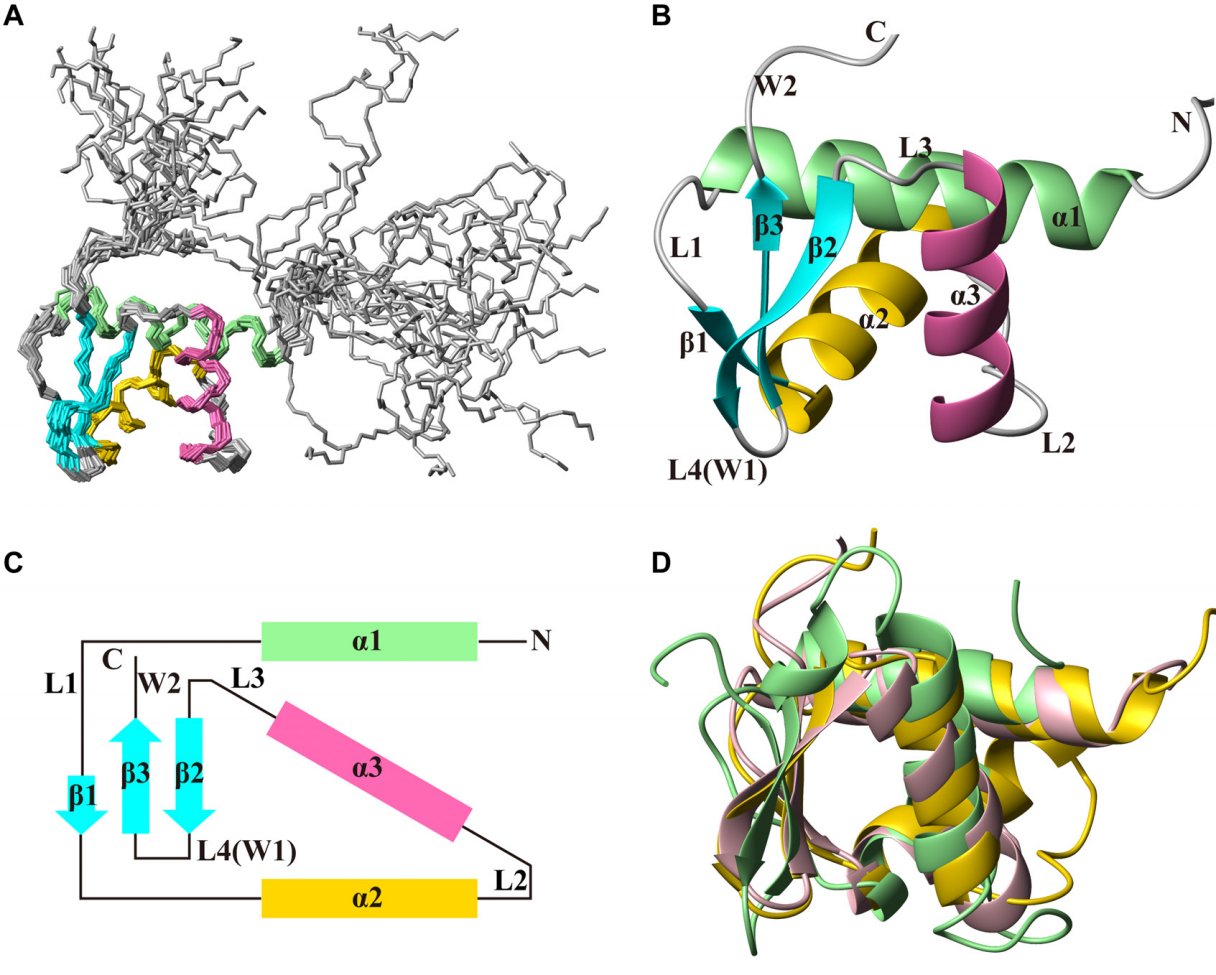
Solution structure of Rok-C^97–191^. PDB code: 5ZUZ. (**A**) Superposition of backbone traces of the ensemble of 20 conformers of Rok-C^97–191^. (**B**) Ribbon representation of the representative structure of Rok- C^97-191^. (**C**) The topology of the structure of Rok-C^97–191^, which belongs to the winged helix family. (**D**) Superposition of the C-terminal domain of Rok (yellow) with winged helix DNA binding proteins PhoB (green, PDB code: 1GXP; RMSD 1.7 Å) and ZBP1 (pink, PDB code: 2LNB; RMSD 1.9 Å).

The solution structure of Rok-C^97–191^ was determined using nuclear magnetic resonance (NMR) spectroscopy (Figure 3A, Supplementary Figure S2, Table 1). Residues 115–179 of Rok-C^97–191^ adopts a typical winged helix fold structure, with an N-terminal tail (residues 97–114) and a C-terminal tail (residues 180–191), both of which are disordered. The winged helix domain has a β-sheet comprising three anti-parallel β-strands (β1, residues 135–136; β2, residues 169–173; β3, residues 176–179), with three α-helices (α1, residues 115–130; α2, residues 137–148; α3, residues 155–165) packed on one side of the β-sheet. The secondary structure elements are arranged in the order of α1-L1-β1-α2-L2-α3-L3-β2-L4-β3 (Figure 3B and C). Based on the nomenclature for winged helix proteins, the L4 loop between β2 and β3 forms wing 1 (W1), while the C-terminal tail forms wing 2 (W2).

**Table 1.** Restraints and structural statistics for Rok-C^97-191^ and Rok-C^102-185^/Seq1 complex

	Rok-C^97–191^	Rok-C^102–185^/Seq1
**Restrains for protein**		
NOE	3078	2542
Intra-residues	858	687
Inter-residues	1046	822
Sequential	452	393
Non-sequential	594	429
Ambiguous	1174	1033
Dihedral angle restraints	110	110
φ angle	57	55
ψ angle	57	55
Chirality restraints	385	351
ω angle	103	92
Chirality	282	259
**Restraints for DNA**		
NOE		983
Hydrogen bonds		62
Sugar pucker		36
Backbone dihedral angle		244
**Protein–DNA intermolecular NOE**		62
Distance restraints violations (>0.2 Å)	0	0
Dihedral angle restraints violations (>5°)	0	0
Average pairwise RMSD (Å)		
Protein (all heavy atoms)	1.04 ± 0.08	0.99 ± 0.19
Protein (backbone heavy atoms)	0.40 ± 0.10	0.44 ± 0.13
DNA (all heavy atoms)		0.69 ± 0.21
Complex (all heavy atoms)		0.71 ± 0.19
**Ramachandran plot**		
Most favored regions (%)	92.8	88.5
Additionally allowed regions (%)	7.2	11.5
Generously allowed regions (%)	0.1	0
Disallowed regions (%)	0	0

A Dali search revealed that the structure of Rok C-terminal domain is highly similar to some winged helix DNA binding proteins, such as PhoB (RMSD 1.7 Å) and the N-terminal domain of human ZBP1 (RMSD 1.9 Å) (Figure 3D) (39,40). PhoB is a transcriptional activator of phosphate genes in *E. coli* which specifically recognizes TGTCA DNA sequence (41), while ZBP1 is an activator of the innate immune response that recognizes Z-DNA (42). The DNA sequence preference of Rok is different from PhoB and ZBP1, as well as any other known winged helix proteins. As the sequence similarities are low between Rok and other winged helix proteins and there is no conserved DNA binding mode for winged helix proteins, the structure of DNA binding domain of Rok in its free form does not reveal its DNA binding mechanism.

A preliminary NMR titration study was carried out with N^15^-labeled Rok-C^97-191^ and a 12 bp DNA d(CGCATATATGCG)_2_ (3AT). The NH signals of many residues were remarkably affected by 3AT DNA, with large chemical shift changes and signal intensity attenuations (Supplementary Figures S3A–C). Residues with large combined chemical shifts differences (Δδ_comb_ = [δ_HN_^2^ + (δ_N_/6.5)^2^]^1/2^) of NH signals were mainly located at the N-terminal regions of the three α-helices, the loop between α2 and α3, and regions around wing W1 (Supplementary Figure S3D). However, the NH signals of some residues at the flexible N- and C-terminal tails were barely perturbed by 3AT DNA, indicating that these residues do not interact with DNA (Supplementary Figure S3D). This was further confirmed by comparing the 2D ^1^H–^15^N HSQC spectra of Rok-C^97–191^ and a shorter construct Rok-C^102–185^ (with residues 97–101 and 186–191 removed) in their free and 3AT DNA bound forms. Except for a few residues close to the truncation sites, most of the NH signals are not affected by the truncation (Supplementary Figures S3E and F). Thus, the short version Rok-C^102–185^ was used for structure determination of the complex in order to simplify NMR spectra.

### Solution structure of Rok-C^102–185^/DNA complex

As the Rok-C^102–185^/3AT complex produced poor triple-resonance NMR spectra, a series of different AT-rich DNA sequences were tested to optimize the qualities of 2D ^1^H–^15^N HSQC (Supplementary Figure S4), 3D HNCACB and 2D F1, F2-^15^N/^13^C-filtered ^1^H–^1^H NOESY spectra. Finally, an 18-bp palindromic DNA d(CTAATAACTAGTTATTAG)_2_ (Seq1) was chosen for structure determination of the complex. Seq1 DNA sequence was designed based on the signature motif AACTA, and we made it palindromic in order to simplify the NMR spectra of DNA.

**Figure 4.**
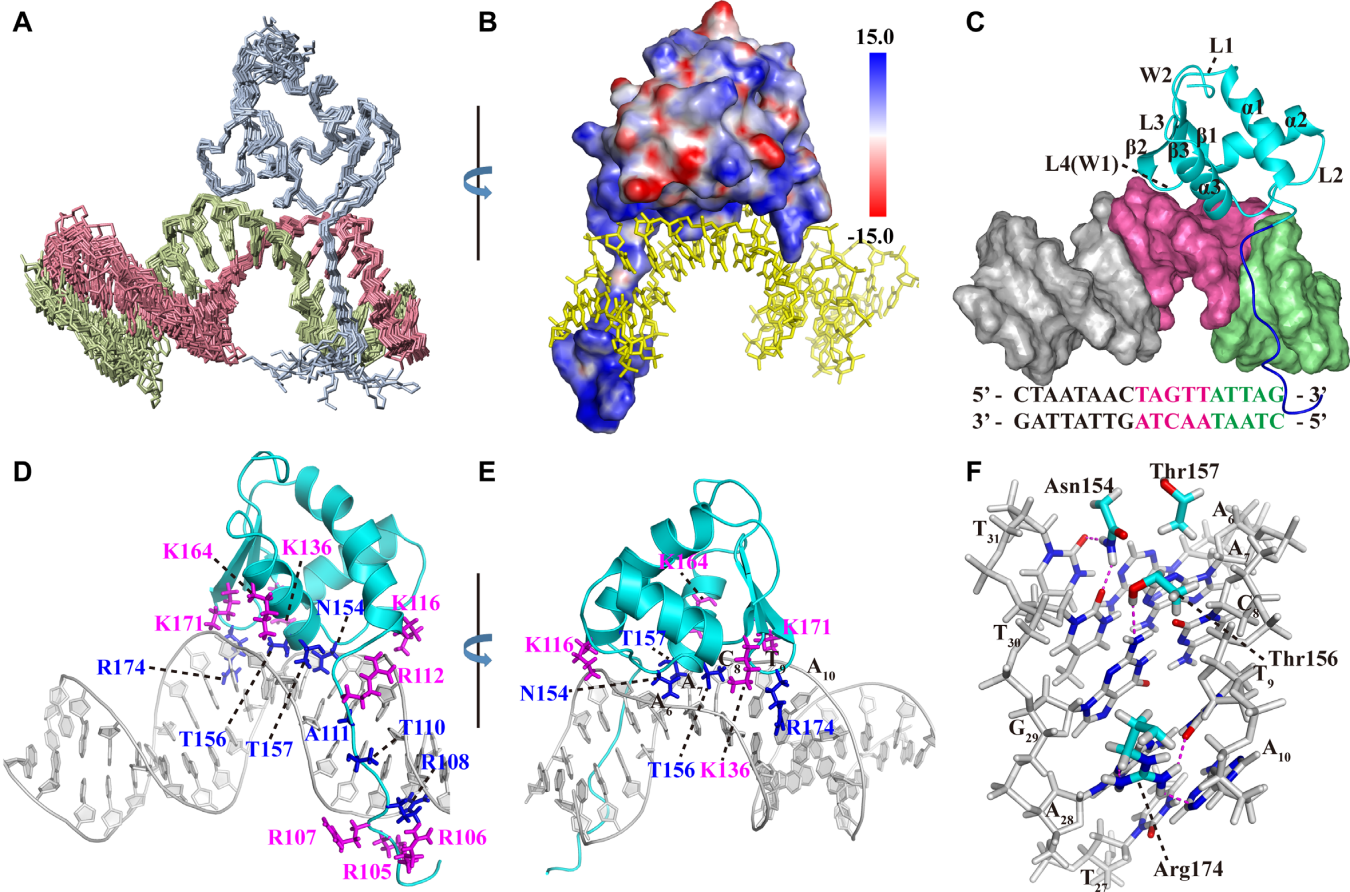
Solution structure of the Rok-C^102-185^/DNA complex. PDB code: 5ZUX. (**A**) Superposition of the protein backbone and DNA for the ensemble of 20 conformers of Rok-C^102–185^ in complex with Seq1 DNA. (**B**) Electrostatic potential surface of Rok-C^102–185^ protein in the complex. (**C**) The winged helix domain (cyan) of Rok binds to the A_6_A_7_C_8_T_9_A_10_ region (hot pink) of Seq1 DNA at the minor groove, while the N-terminal tail (blue) interact with the C_1_T_2_A_3_A_4_T_5_ region (pale green). (**D** and **E**) Protein residues interacting with Seq1 DNA. Residues inserted into the minor groove are colored blue and residues interacting with the phosphate groups are colored magenta. (**F**) Interactions of N154, T156 and R174 with the bases of the A_6_A_7_C_8_T_9_A_10_ region in the minor groove through hydrogen bonds.

The solution structure of the Rok-C^102–185^/Seq1 complex was determined based on 2604 intra-protein NOE signals, 991 intra-DNA NOE signals, and 62 intermolecular NOE signals (Figure 4A). Resonance assignments of the 2D F1, F2-^15^N/^13^C-filtered ^1^H–^1^H NOESY, 2D ^1^H–^15^N HSQC and 3D F1-^15^N/^13^C filtered, F2-^13^C edited NOESY-HSQC are shown in Supplementary Figure S5. Restraints and structural statistics are summarized in Table 1.

Rok-C^102-185^ binds the minor groove of Seq1 DNA and the binding surface is positively charged (Figure 4B). It appears that the protein-DNA interface consists of two parts (Figure 4C). The N-terminal tail interacts with the C_1_T_2_A_3_A_4_T_5_ region, with residues R108, G109, T110 and A111 positioned into the DNA minor groove and residues R105, R106, R107 and R112 interacting with the phosphate groups of the DNA (Figure 4D). The winged helix domain (residues 115–179) mainly binds at the A_6_A_7_C_8_T_9_A_10_ region, with sidechains of residues N154, T156, T157 and R174 positioned in the DNA minor groove, while four lysine residues K116, K136, K164 and K171 interacting with DNA backbone phosphate groups (Figure 4E).

An N-terminal tail truncated construct Rok-C^114–185^ was used to parse the roles of the N-terminal tail and the winged helix domain of Rok in DNA binding. Comparison of the 2D ^1^H–^15^N HSQC spectra of Rok-C^114–185^ and Rok-C^102–185^ in either free or DNA bound state revealed that most of the NH signals of Rok-C^114–185^ are coincident with those of Rok-C^102–185^ except for a few residues near the truncation site (Supplementary Figures S6A and S6B), indicating that the removal of N-terminal tail does not affect the DNA binding properties of the winged helix domain. However, isothermal titration calorimetry (ITC) measurements revealed that the binding affinity of Rok-C^114–185^ to Seq1 DNA (*K*_d_ = 19.7 ± 2.7 μM) is slightly lower than that of Rok-C^102–185^ (*K*_d_ = 7.9 ± 1.3 μM) (Supplementary Figure S6C, Table 2). Taken together, the N-terminal tail does contribute to DNA binding, but its role is independent of the winged helix domain.

**Table 2.** Equilibrium dissociation constants of Rok-C^102–185^, Rok-C^114–185^ and its mutants binding to Seq1 DNA, measured using ITC. The uncertainties indicate the combined standard deviations of two measurements (N/D: not detectable)

	*K*_d_ (μM)
Rok-C^102–185^	7.9 ± 1.3
Rok-C^114–185^	19.7 ± 2.7
R174A	89 ± 42
T156A	42.9 ± 7.1
T157A	15.0 ± 1.9
N154D	N/D
K171A	110 ± 35
K116A	42.9 ± 8.1
K136A	39.4 ± 6.4
K164A	30.9 ± 2.9

The ITC data also indicated that, the binding processes of both Rok-C^102–185^ and Rok-C^114–185^ with Seq1 DNA are endothermic and entropy-driven, which are characteristics of DNA minor groove binding (43). The binding to minor groove by Rok is further confirmed by NMR competition experiments using netropsin, an AT-rich DNA minor groove binding drug (44). NH signals of DNA-bound Rok-C^114–185^ shifted towards the positions of the free protein in the 2D ^1^H–^15^N HSQC spectra when netropsin was gradually titrated into the sample, an indication of protein releasing from the DNA (Supplementary Figure S7A).

It was previously reported that the binding of Rok with *PcomK* promoter DNA is inhibited by the DNA major groove binding drug methyl green, but not the DNA minor groove binding drug chromomycin A3 (27). As chromomycin A3 targets the minor groove of GC-rich DNA (45), it is reasonable that chromomycin A3 lacks the ability to interfere with the binding of Rok with AT-rich DNA. To explain why methyl green inhibits Rok binding DNA, we tried an NMR competition titration experiment with methyl green. It was found that methyl green can cause precipitation of the Rok-C^114–185^/Seq1 complex, leading to uniform attenuation of the 2D ^1^H–^15^N HSQC signals (Supplementary Figure S7B). Moreover, Rok-C^114–185^ can also be precipitated by the addition of methyl green (Supplementary Figure S7C). Therefore, the previous observed inhibitory effect of methyl green on the binding of Rok with *PcomK* most likely results from its propensity to precipitate Rok protein, rather than a direct inhibition of DNA binding *per se*.

Comparing the structure of the winged helix domain in the complex with that of the free from, it is obvious that DNA binding results in some notable conformational changes. While the conformation of the β-sheet mostly remains the same, the N-terminal region of α1 helix bends towards L2 loop, causing the α3 helix and L2 loop to move closer to the β-sheet and the separation between α2 and α3 helices to become wider (Supplementary Figure S8A). These conformational changes are consistent with the large differences between the 2D ^1^H–^15^N HSQC spectra of Rok-C^102–185^ in its free and Seq1 DNA bound forms (Supplementary Figure S8C). In addition to the protein residues at the binding interface, the NH signals of many other residues are also significantly perturbed by DNA binding (Supplementary Figure S8B), and the chemical shifts of many methyl groups are also significantly changed (Supplementary Figure S8D).

**Figure 5.**
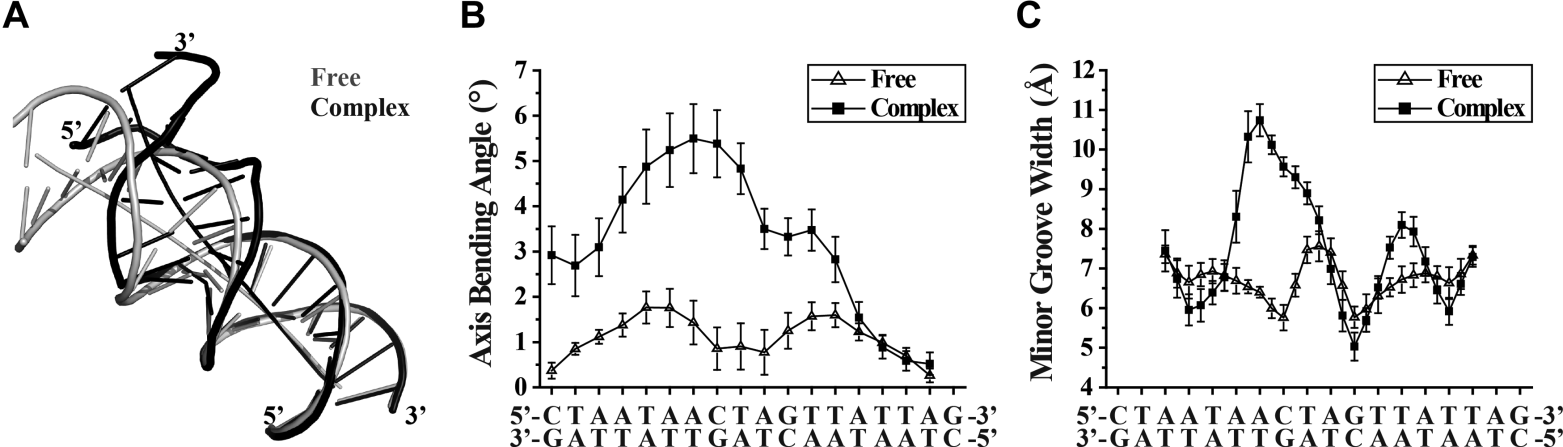
Conformational changes of Seq1 DNA upon binding with Rok-C^102–185^. (**A**) Superimpositions of mean structures of Seq1 DNA in Rok-C^102–185^ bound (black) and free (gray) forms. (**B** and **C**) Changes of the axis bending angles and minor groove width of Seq1 DNA upon Rok binding (calculated using Curves+).

Rok binding also leads to a large conformational change of Seq1 DNA (Figure 5A). Rok-bound DNA is bent ∼25 degrees due to the protein binding (Figure 5B), and the minor groove of the A_6_A_7_C_8_T_9_A_10_ region is significantly widened, likely as a result of the insertion of sidechains of residues N154, T156, T157 and R174 (Figure 5C). This may explain why Rok prefers TpA steps to A-tracts (Figure 1C and D), as the TpA step is the most conformationally flexible of all the ten possible base-steps (46), and it has a tendency to widen the minor groove of AT-rich DNA. On the contrary, A-tract DNA is rigid and has a narrow minor groove (47).

### Mutagenesis of residues at binding interface of winged helix domain

In the structural ensemble of Rok-C^102–185^/Seq1 complex, several hydrogen bonds between DNA bases and sidechains of the winged helix domain residues could be observed in some of the structures. The guanidine group of R174 may form hydrogen bonds with the N3 atom of A_28_ (17/20, 17 out of 20 structures), the O2 atom of T_9_ (14/20), and/or the N3 atom of A_28_ (9/20). The NH_2_ group of N154 may form hydrogen bonds with the O2 atoms of T_30_ (18/20) and/or T_31_ (20/20), and the OH group of T156 may form a hydrogen bond with the N2 atom of G_29_ (19/20) (Figure 4F). In addition, the positively charged sidechains of residues K116, K136 and K164 of the winged helix domain are positioned close to phosphate groups of A_32_, T_30_, and C_8_, respectively, suggesting they contribute to Rok binding via electrostatic interactions with the phosphate backbone. Likewise, the sidechain of residue K171 appears to interact with the phosphate groups of both T_9_ and A_10_ (Figure 4E).

Mutagenesis studies were carried out to further evaluate the roles of these residues of Rok in DNA binding. The *K*_d_ values of Rok-C^114–185^ mutants with Seq1 DNA are summarized in Table 2. The R174A mutant of Rok-C^114–185^ binds Seq1 DNA with a *K*_d_ value of 89 ± 42 μM, about 4.8-fold of wild-type (WT) Rok-C^114–185^ (19.7 ± 2.7 μM) (Supplementary Figure S11A), and the scale of chemical shift perturbations on R174A caused by Seq1 DNA is significantly attenuated compared with WT protein (Supplementary Figure S10A), indicating that R174 is a key residue for DNA binding. Mutation of T156 to Ala also weakens DNA binding as measured by ITC (*K*_d_ = 42.9 ± 7.1 μM) (Supplementary Figure S11B), and observed through decreased chemical shift perturbations (Supplementary Figure S10B). Mutating T157 to Ala has little influence on the DNA binding affinity (*K*_d_ = 15.0 ± 1.9 μM) (Supplementary Figure S11C), and no significant difference exists in the chemical shift perturbations (Supplementary Figure S10C). The N154A mutant protein is unfolded (Supplementary Figure S9A), perhaps due to a loss of a hydrogen bond between the sidechain carbonyl group of N154 and the backbone NH group of T157 (Supplementary Figure S9D), which may contribute to the structural stability of the protein. This is supported by the finding that mutants N154S and N154Q are also unfolded (Supplementary Figures S9B and S9C), while N154D mutant is well folded similar to the WT protein as the substitution of Asn by Asp should be able to retain the hydrogen bond (Supplementary Figure S10D), yet the ITC isotherm of the N154D mutant with Seq1 DNA indicates it binds DNA poorly and the *K*_d_ value is difficult to measure (Supplementary Figure S11D). Moreover, the chemical shift perturbations of Seq1 DNA on N154D are much smaller compared with the WT protein (Supplementary Figure S10D).

K171A mutation significantly weakens DNA binding. The *K*_d_ value of K171A mutant with Seq1 DNA is 110 ± 35 μM (Supplementary Figure S11E), about 5.6-fold higher than that of the WT, and the chemical shift perturbations of Seq1 DNA on protein NH signals are also largely attenuated (Supplementary Figure S10E). K171 is located on the β2 strand near wing W1 and interacts with the phosphate groups of T_9_ and A_10_, while wing W1 residue R174 interact with the bases of this TpA step. These two residues may act synergistically to stabilize the binding between wing W1 and Seq1 DNA. The *K*_d_ values of K116A, K136A, and K164A mutants with Seq1 DNA are 42.9 ± 8.1, 39.4 ± 6.4 and 30.9 ± 2.9 μM (Supplementary Figures S11F–H), which increased ∼1.7–2.4-fold compared with the WT Rok^114–185^ protein. These mutants show only slight attenuation in the chemical shift perturbations caused by Seq1 DNA (Supplementary Figures S10F–H).

Taken together, the structure of the complex and these mutagenesis studies show that residues N154, K171 and R174 are most important for DNA binding, while residues K116, K136, T156 and K164 make smaller contributions.

### DNA sequence recognition specificity by Rok

**Figure 6.**
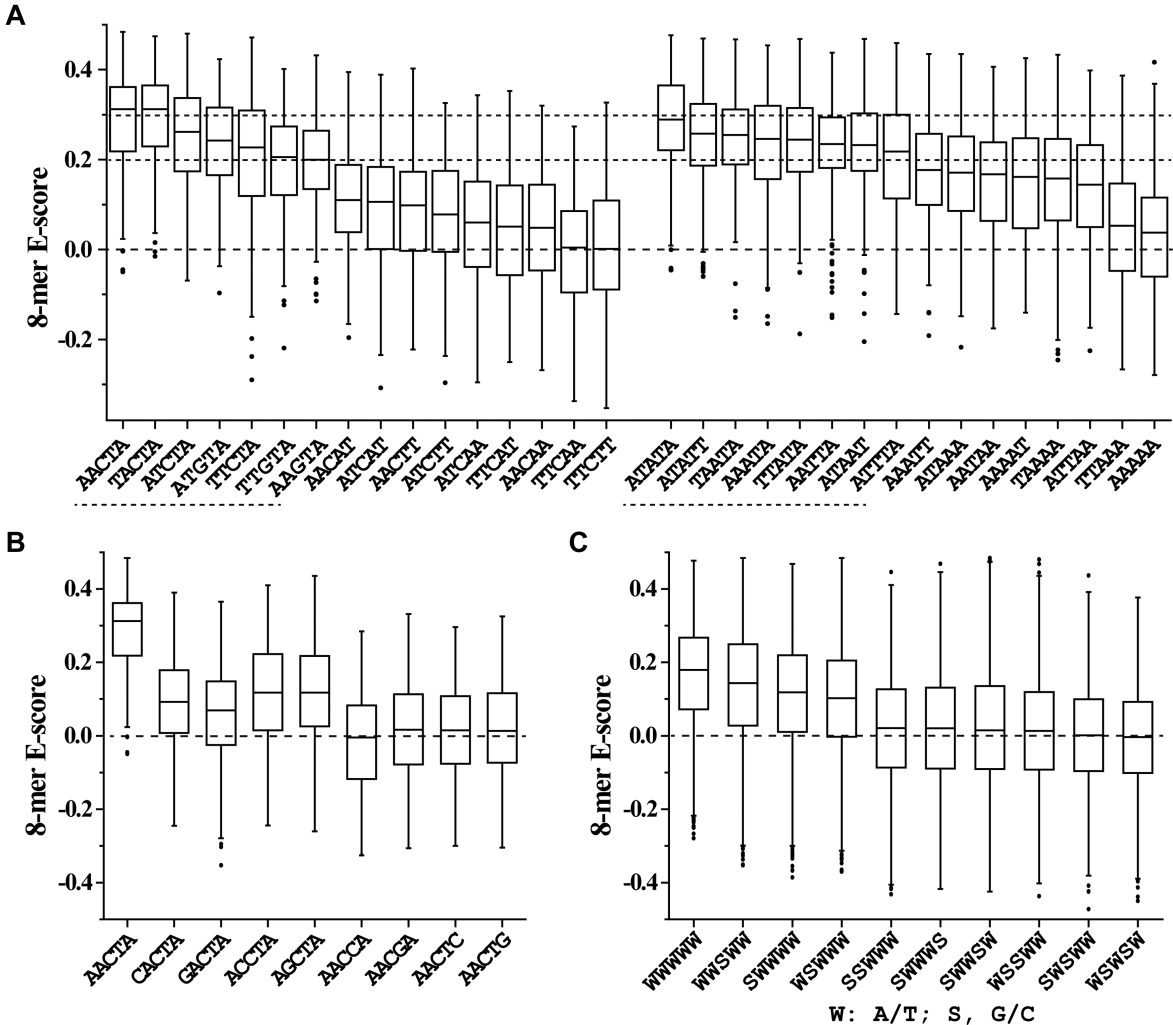
DNA binding preferences of Rok towards different 5-bp sequences. The distribution of *E*-scores of all 8-mers containing a specific 5-bp sequence or its complementary sequence is presented using box plot. (**A**) DNA binding preference of Rok toward 5-bp sequences containing only one GC base pair in the middle or containing only AT base pairs. Sequences with median E-scores above 0.20 are underlined with dashed lines. The tops three sequences are in bold. (**B**) Comparison of AACTA with its variant sequences that contain an extra GC base pair. (**C**) The influence of G/C insertion positions in AT-rich 5-bp sequences.

In the light of the structural data, we reexamined the PBM experiments to better explain how Rok may preferentially target specific DNA sequences. Our structure revealed that the winged helix domain of Rok covers a region of ∼5 bp on Seq1 DNA. We therefore compared the *E*-scores of 8-mers containing different 5-bp sequences. Among all 8-mers, those containing the 5-bp sequences AACTA and TACTA are the most favored (median *E*-score > 0.30), followed by ATATA which may represent multiple adjacent TpA steps (Figure 6A). Consistently, for the 27 8-mers with *E*-scores above 0.45, 14 of them contain AACTA or TACTA, 4 of them contain ATCTA or TTCTA, and the other 9 8-mers contain at least three TpA steps. These results indicate that Rok indeed has a higher preference for these specific sequences. Sequences where any of the four AT base pairs of AACTA is replaced by a GC base pair showed significantly lower *E*-scores (Figure 6B). More generally, 5-bp sequences with two or more GC base pairs are disfavored. And for 5-bp sequences containing one GC base pair, sequences are more preferred if the GC base pair is in the middle (Figure 6C).

The central location of the GC-base pair is consistent with the finding that N154 and R174 insert deeply into the DNA minor groove and that they each interact with consecutive AT base pairs separated by one base pair. Compared with AT base pairs, GC base pairs contain an exocyclic 2-NH_2_ group that protrudes in the minor groove, which may sterically prevent the insertion of N154 and R174 in the minor groove if the inserted GC base pair is not at the middle of the 5-bp sequence. The higher affinity of Rok for sequences with a centrally located cytosine residue (AACTA and TACTA) compared to similar sequences without one (e.g. AAGTA, AATTA, AAATA and TAATA) is consistent with the observation that that there is likely a hydrogen bond between the OH group of T156 and the N2 atom of the central guanine on the complementary chain (and that mutation of T156 to Ala weakens binding affinity).

For 5-bp sequences containing no GC base pairs, ATATA is the most preferred sequence, which may be due to its multiple adjacent TpA steps. ATATT, TTATA, TAATA, AAATA and AATTA are slightly less preferred sequences, which differ by only a single-base-pair from ATATA, TACTA or AACTA. The A-tract sequence AAAAA is the most disfavored. Generally, sequences containing no TpA steps (e.g. AACAA, AACTT or AACAT) are poor targets for binding by Rok; they might be inappropriate in shape and lack enough flexibility to accommodate Rok binding. Consistent with this notion, the DNA binding ability of the winged helix domain to Seq1 DNA is significantly attenuated when either K171 or R174, which contact the T_9_A_10_ step, is changed to alanine.

The finding that N154 binds the AA•TT base pairs of AACTA via two hydrogen bonds with the O2 atoms of the two thymine bases may explain why AACTA is favored over ATCTA and TTCTA. Rok has similar affinities for TACTA and AACTA possibly because the additional TpA step increases the flexibility of DNA and compensates for the loss of a hydrogen bond.

To further investigate how Rok interacts with TpA steps we used another DNA molecule d(CATATATATATATATATG)_2_ (8AT) as a binding substrate. The 2D ^1^H–^15^N HSQC spectrum of Rok-C^102-185^/8AT was compared with Rok-C^102–185^/Seq1. Most NH signals are only slightly changed (Δδ_comb_ < 0.05 ppm), indicating that the binding mode of Rok to ATATA is similar to AACTA. Residues with combined chemical shift differences of NH signals larger than 0.10 ppm are S138, Q141, K142 and N154-Q160 (Supplementary Figures S12A and B). This might be a reflection of the fact that in Rok-C^102–185^/Seq1 complex structure, these residues all lie near the ApC step of AACTA, which changes to TpA for 8AT DNA (Supplementary Figure S12C). In comparison, the NH signals of residues near the TpA step shared by AACTA and ATATA have very small changes (Δδ_comb_ < 0.05 ppm) (Supplementary Figure S12C).

## DISCUSSION

### Rok adopts a DNA binding mechanism different from the other xenogeneic silencers

**Figure 7.**
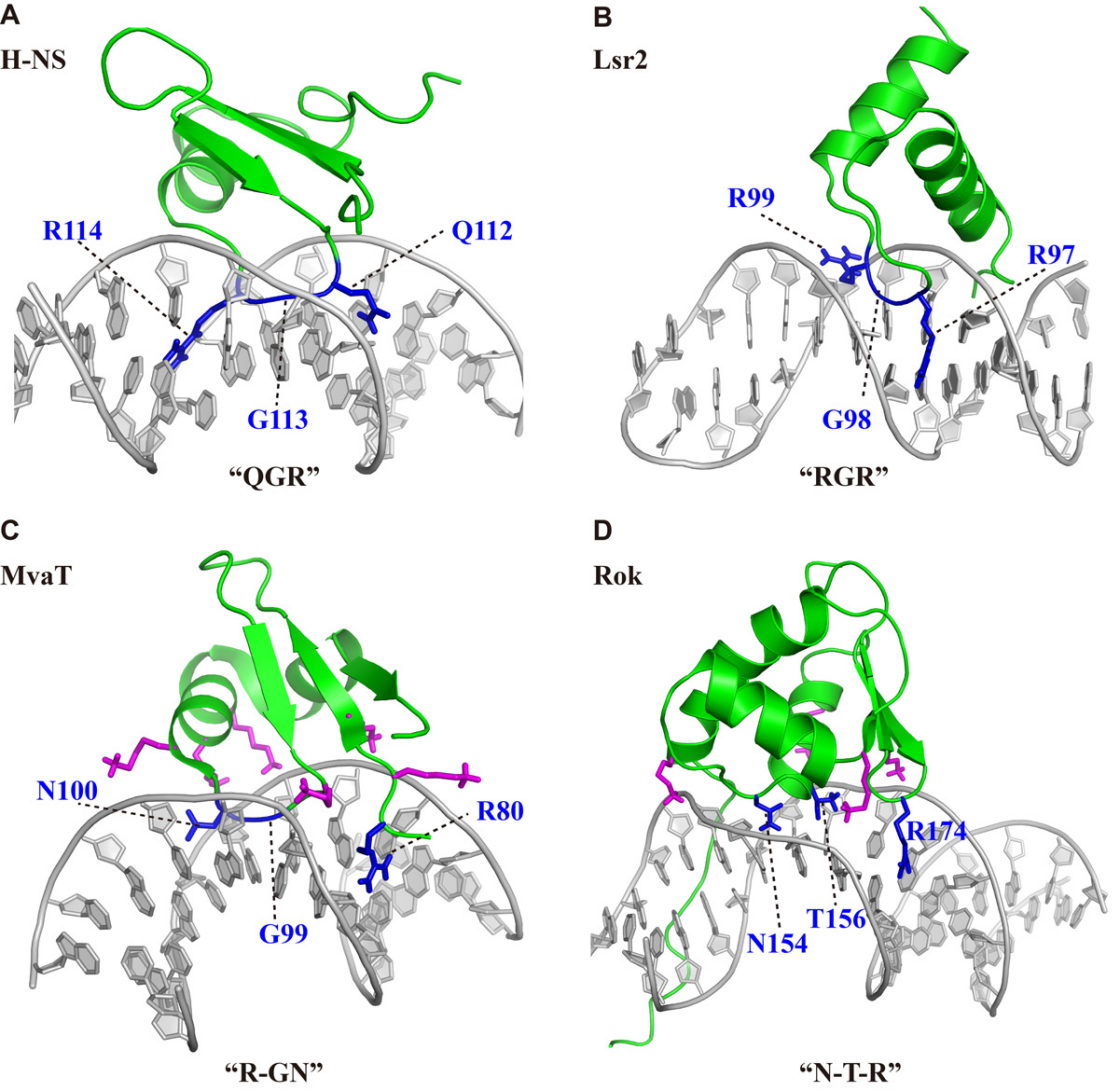
Comparison of the DNA recognition mechanisms of four types of xenogeneic silencers. (**A**) Structure model of the H-NS DNA-binding domain in complex with 3AT DNA (13). The ‘AT-hook-like’ structure ‘QGR’ are colored blue. (**B**) Structure model of Lsr2 DNA-binding domain in complex with a 27 bp hairpin DNA CCTAATTATAACGAAGTTATAATTAGG (13). The ‘AT-hook-like’ structure ‘RGR’ are colored blue. (**C**) Structure of MvaT DNA-binding domain in complex with 3AT DNA (PDB code: 2MXF). The ‘AT-pincer’ structure ‘R-GN’ are colored blue and the six lysine residues interacting with the sugar-phosphate backbones are colored magenta. (**D**) Structure of Rok DNA-binding domain in complex with Seq1 DNA. The three residues ‘N-T-R’ involved in the formation of intermolecular hydrogen bonds in the DNA minor groove are colored blue, and the four lysine residues interacting with the phosphate groups are colored magenta.

Among the four kinds of known xenogeneic silencers (H-NS, Lsr2, MvaT and Rok), Rok was the last one identified. We have previously reported that H-NS and Lsr2 bind AT-rich DNA sequences through a conformationally conserved ‘AT-hook-like’ motif composed of three continuous residues ‘Q/RGR’, although the structures of their DNA binding domains are distinct. This binding is sterically blocked by the extracyclic amino group of guanine bases and hence these proteins show a strong preference for short sequence motifs of consecutive AT base pairs (Figure 7A and B) (13,14). Differences between H-NS and Lsr2 are that Lsr2 prefers A-tracts, while flexible TpA steps are generally more preferred by H-NS. The overall fold of the DNA binding domain of MvaT is similar to that of H-NS, but it uses three non-consecutive residues ‘R-GN’ that form an ‘AT-pincer’ structure to recognize AT-rich DNA, leaving a cavity in the protein/DNA interface, which may endow MvaT with a higher tolerance for G/C interruptions in its binding site compared to H-NS and Lsr2 (Figure 7C). Meanwhile, six lysine residues interact electrostatically with the DNA backbone phosphate groups and assist the binding. Binding of MvaT to DNA leads to conformational changes of the DNA molecule. Rigid A-tracts are not favored and the absence of TpA steps from a given sequence imparts a higher penalty on binding of MvaT than it does on H-NS or Lsr2 (15).

The DNA binding domain of Rok adopts a winged helix fold, which is totally different from the other three xenogeneic silencers. Rok does not contain any ‘AT-hook-liker’ or ‘AT-pincer’ structure, and it recognizes the DNA minor groove through three non-consecutive residues ‘N-T-R’, assisted by four lysine residues interacting with the phosphate groups (Figure 7D). Sidechains of residues N154 and T156 around the N-terminal region of α3 helix, as well as residue R174 of wing W1, can form hydrogen bonds with bases inside the minor groove. This DNA recognition mechanism enables Rok to prefer some AT-rich DNA sequences with G/C insertion, such as TACTA and AACTA, while the other three xenogeneic silencers all prefer sequences with 100% AT-content, although MvaT has a better G/C base insertion tolerance. The bias of Rok for TpA steps, as compared to A-tracts, is even more significant than what is observed for MvaT. Correspondingly, Rok induces larger conformational changes on its DNA target than MvaT. More remarkably, Rok has a high preference for some specific motifs, while no significant sequence specificity has been found for H-NS, Lsr2 or MvaT (14,15).

### Rok shows a novel DNA binding mode for winged helix proteins

**Figure 8.**
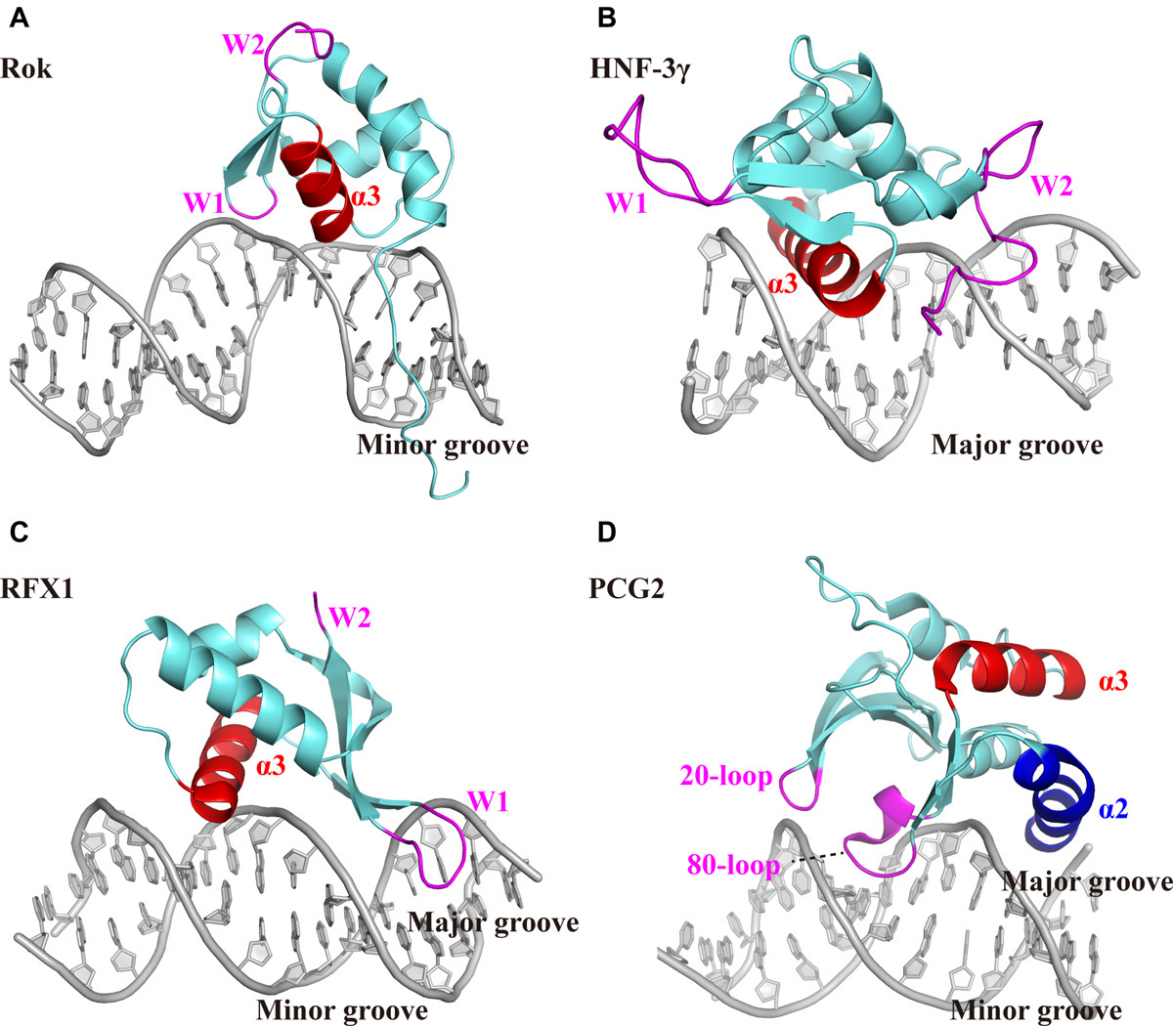
Comparison of the DNA binding mode of Rok with other winged helix proteins. The α3 helix are colored red and the wings are colored magenta. (**A**) The winged helix domain of Rok binds the minor groove of DNA with its wing W1 and α3 helix. (**B**) HNF-3γ binds the major groove of DNA with its α3 helix, assisted by wing W1 and W2 (PDB code: 1VTN). (**C**) RFX1 mainly binds the major groove with its wing W1, assisted by α3 helix contacting the minor groove (PDB code: 1DP7). (D) The DNA binding domain of PCG2 adopts an atypical winged helix fold, with two wings termed 20-loop and 80-loop binding the minor groove and α2 helix interacting with the major groove.

The winged helix domain is a widespread nucleic acid binding domain. Recently, Liu *et al.* have classified the DNA binding modes of winged helix proteins into three types (48). In the typical DNA binding mode, represented by HNF-3γ, winged helix proteins use the α3 helix to recognize and bind DNA major groove, while wings W1 and W2 assist the binding by interacting with the minor groove or the sugar-phosphate backbone (Figure 8B) (48). This DNA binding mode is also found in E2f/DP2 (49), LexA (50), CAP (51), ETS (52) and some other winged helix proteins. Another type is found in RFX1 (53), which mainly uses its wing W1 to interact with the DNA major groove, while the α3 helix of RFX1 plays an auxiliary role by interacting with the minor groove (Figure 8C). A third DNA binding mode is represented by the DNA binding domain of PCG2, which adopts an atypical winged helix fold with four helices (48). PCG2-DBD uses two wing structures termed 20-loop and 80-loop to bind the minor groove, while its α2 helix preceding 80-loop interacts with the major groove (Figure 8D).

Rok is the first identified winged helix protein which only interacts with the minor groove of DNA, even though it adopts a typical winged helix domain fold. Like many other winged helix proteins, the α3 helix and following wing W1 of Rok play critical roles in DNA binding. However, they both make contact with the minor groove of DNA (Figure 8A), which is significantly different from the previously classified three types of DNA binding modes for winged helix proteins, indicating that the way winged helix proteins bind DNA could be more versatile.

### The DNA binding mechanism is conserved among Rok family proteins

Previous studies have found several Rok-like proteins in *B. subtilis, B. amyloliquefaciens, B. coagulans, B. licheniformis, B. atrophaeus* and *B. pumilus* species, using the Blastp program with Rok from the *B. subtilis* subsp. subtilis str. 168 as a seed (49). Singh *et al.* found that a conjugative *B. subtilis* plasmid pLS20 encodes a Rok homologue Rok_LS20_ with a considerably shorter sequence. Blast and Psi-Blast searches using Rok_LS20_ as the query sequence produced 20 Rok orthologues in the *Bacillus* genus, which were classified as ‘large Rok proteins’ and ‘small Rok proteins’ based on their length and genomic loci of their encoding genes (50).

We used Blastp to search the sequence of Rok-C^114-185^ against the non-redundant database of NCBI, and uncovered 201 Rok homologous sequences corresponding to ∼800 GeneBank protein items, with identities ranging from 33% to 100% (supplementary dataset S2). These proteins are mainly found in the *Bacillus* genus, with a few from *Domibacillus, Jeotgalibacillus* and some other isolates (Supplementary Figure S13).

**Figure 9.**
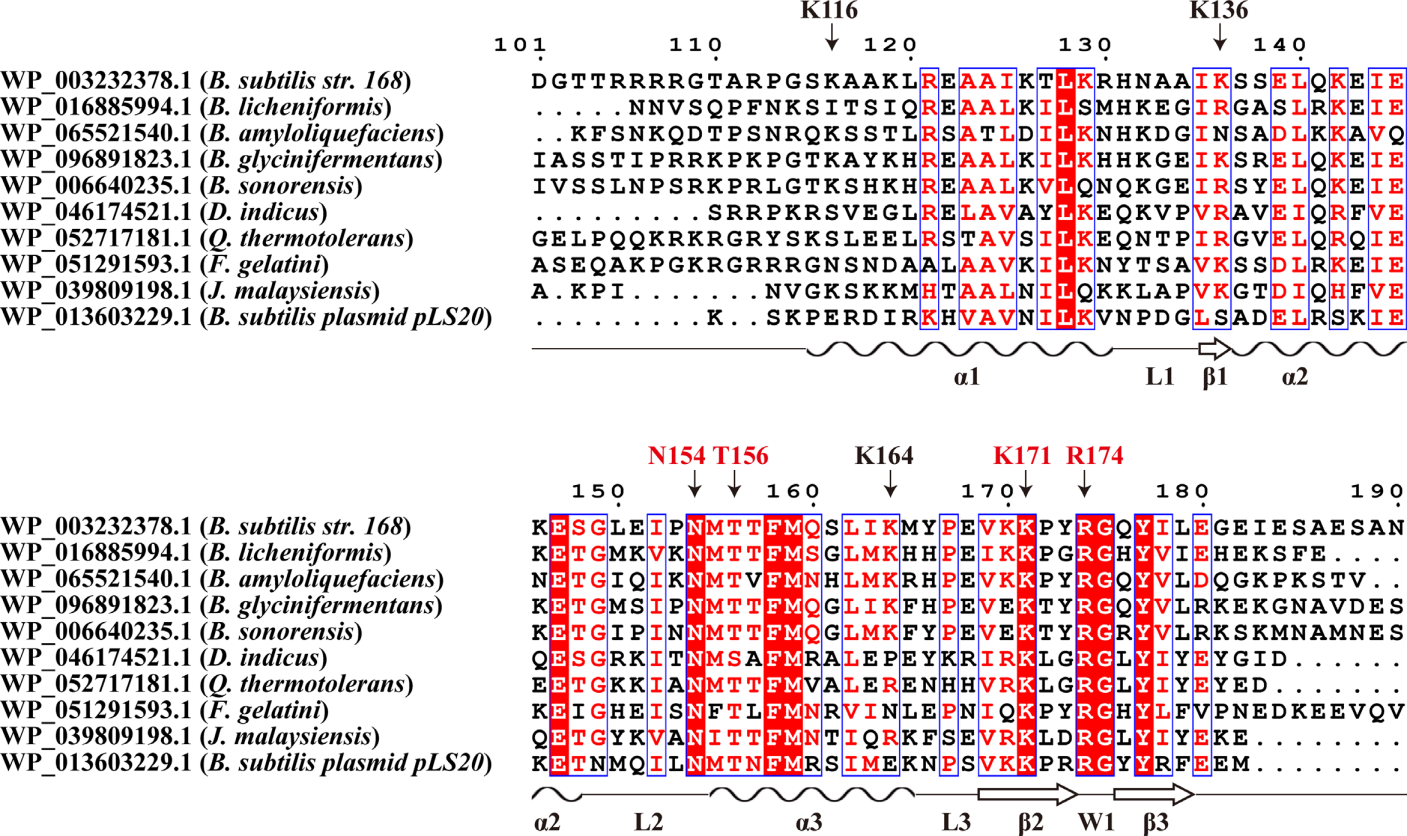
Sequence alignment of the C-terminal domains of Rok homologs. Sequences are aligned using Clustal Omega and the image is generated using ESPript. Residues important for DNA binding are indicated.

A sequence alignment of the C-terminal domains of representative Rok homologs is shown in Figure 9. The three α-helices, three β-strands, L2 loop and wing W1 are conserved regions, while the sequences of L1 loop, L3 loop and the C-terminal tail are variable. The key residues in DNA binding N154, K171 and R174 are invariant whereas T156 is highly conserved. The three auxiliary residues K116, K136 and K164 at the interface are less conserved. The N-terminal tail is not conserved, but often contains some positively charged residues which should assist DNA binding. These findings suggest that all Rok homologous proteins should share the same DNA binding mechanism, regardless of the sequence identities.

### The DNA binding preference of Rok relates to the characteristics of *B. subtilis* genome

Xenogeneic silencers must not only target foreign DNA but must avoid accidentally targeting self DNA. The subtle differences in the DNA binding preference of xenogeneic silencers may have evolved to enable each of these silencers to efficiently recognize certain sequences/structures as foreign against the backdrop of their particular ‘self’ genomes. (15). For example, the AT-content of *Mycobacteria tuberculosis* genome (∼34%) is lower than *Samonella enterica* (∼48%). Correspondingly, Lsr2 from *M. tuberculosis* can recognize some foreign genes with a relatively lower AT-content compared with H-NS from *S. enterica*, as the DNA binding ability of Lsr2 mediated by ‘RGR’ is relatively higher than H-NS mediated by ‘QGR’ (14). *Pseudomonas aeruginosa* also has a genome with low AT-content (∼33%). The higher tolerance of MvaT to G/C insertions may help MvaT inhibit more foreign genes with relatively lower AT-content (15).

**Figure 10.**
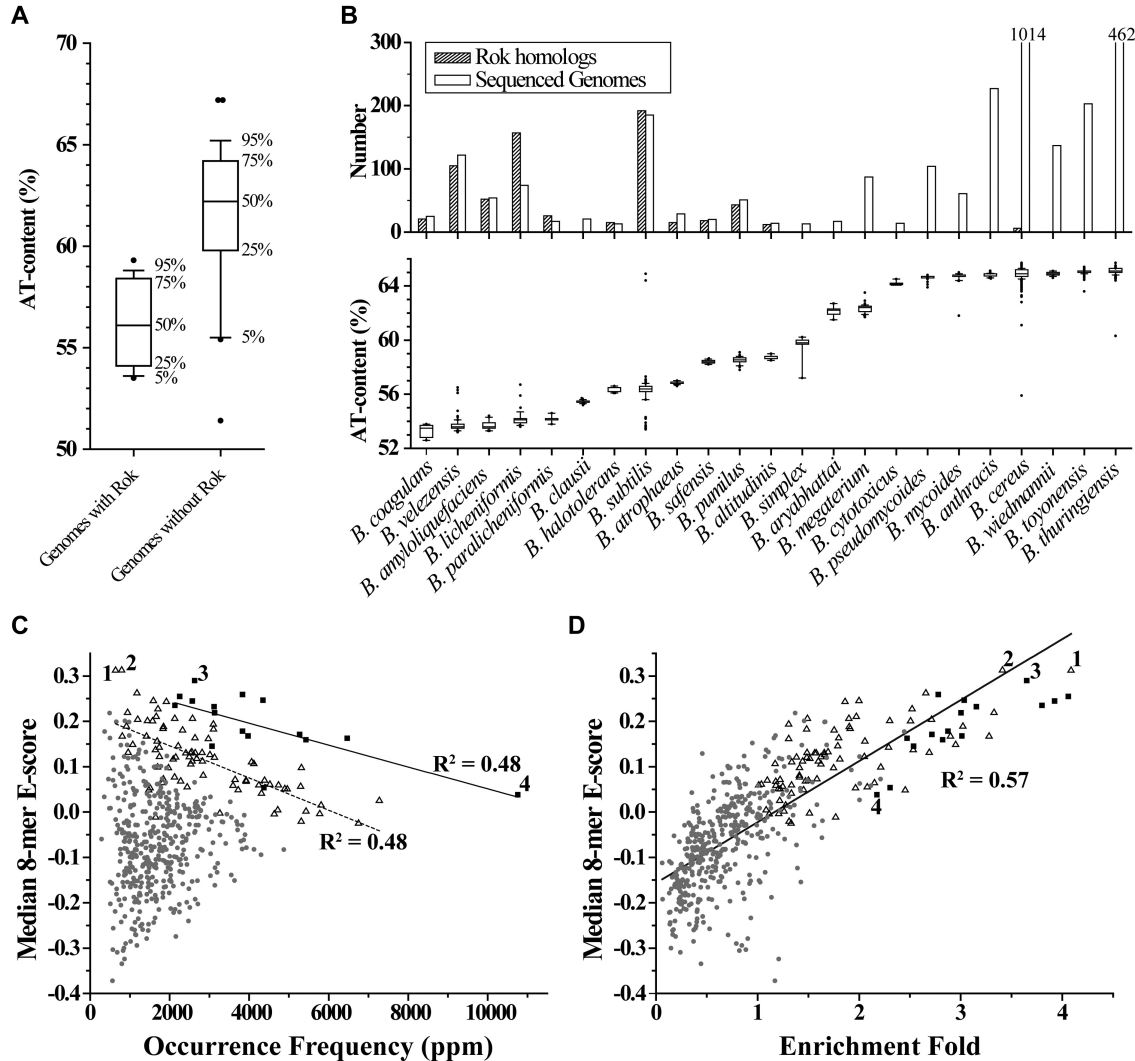
The characteristics of bacterial genomes that contain Rok homologs. For the box plots here, bands at the bottom, top, and inside of the box represent the first quartile, the third quartile, and the median, respectively. The whiskers indicate points within 5%∼95% range, and the black dots represent the outliers. (**A**) AT-contents of *Bacillus* genomes with/without Rok homologs. (**B**) AT-contents of the sequenced genomes for each individual *Bacillus* species and the number of Rok homologous proteins found in each specie. *Bacillus* species with ≥ 10 sets of genomic sequence data in the NCBI database are presented. (**C**) The genome occurrence frequency of each 5-bp sequence is plotted against the median 8-mer E-score from the PBM data. The occurrence frequency of a certain kind of 5-bp sequence was calculated in the unit of parts per million (ppm) by adding the occurrence counts of both itself and its complementary sequence together and then divided by the length of the genome. The solid and dashed lines are the linear fitting for the data of 5-bp sequences 5 or 4 A/T, respectively. (**D**) Rok binding regions from the ChIP-seq data by Seid *et al.* were extracted from the genome and each was extended to 300 bp centered by the 40 bp binding peak. The enrichment fold of each 5-bp sequence was calculated via dividing its occurrence frequency in Rok binding regions by that in the *Bacillus* genome and was plotted against the median 8-mer E-score. The solid line is the linear fitting for the data of all 5-bp sequences. Black squares (▪), hollow triangles (–), and small grey dots (•) represent 5-bp sequences with 5, 4, and ≤3 A/T, respectively. 1, TACTA; 2, AACTA; 3, ATATA; 4, AAAAA.

Different from the genomes referred above, almost all the *Bacillus* genomes are AT-rich with AT-contents ranging from 51% to 68%, except for a few. Interestingly, we found that the majority of *Bacillus* species that contain Rok homologs have a genomic AT-content between 53% and 59%, such as *B. velezensis, B. amyloliquefaciens, B. licheniformis, B. subtilis*, and *B. pumilus*. In comparison, most of *Bacillus* species without Rok often show a significantly higher genomic AT-content (> 59%), such as *B. megaterium, B. pseudomycoides, B. anthracis, B. cereus, B. wiedmannii, B. toyonensis*, and *B. thuringiensis* (Figure 10A and B, supplementary dataset S2). This suggests that those genomes with relatively lower AT-contents should have some common features characterized for foreign genes, which can be recognized by Rok.

As we have found that Rok has a higher preference for some specific sequences, especially for TACTA, AACTA, and ATATA (Figure 6A), we calculated the occurrence frequency of each 5-bp sequence in the genome of *B. subtilis subsp. subtilis str. 168* (NC_000964.3) (supplementary dataset S3). The results showed that the core genome is significantly depleted for the AT-rich motifs preferred by Rok, although it has a high AT-content of 56.5%. For 5-bp sequences with 4 or 5 A/T, the occurrence frequencies are negatively correlated with the corresponding median 8-mer E-scores of the PBM data. Strikingly, TACTA and AACTA, which have the highest median 8-mer E-scores among all 5-bp sequences, are the least represented sequences in *B. subtilis* genome among all 5-bp sequences with 4 or 5 A/T, ranking 493 and 473 among all 512 5-bp sequences, respectively. In addition, there is a dramatic over-representation of A-tract sequences and a relative under-representation of sequences with consecutive TpA steps. For example, the occurrence frequency of sequence AAAAA is about four times higher than that of the sequence ATATA (Figure 10C). These findings indicate that the *B. subtilis* core genome is strongly biased against the DNA sequence motifs which Rok most avidly binds (e.g. TACTA), which should reduce adventitious Rok binding to ‘self’ sequences. Conversely we would predict that the sequences bound by Rok will display compositional biases that differ from those of the core genome. To test this, the occurrence frequencies of 5-bp sequences in the Rok binding sites mapped by Seid *et al.* using ChIP-seq were analyzed (24), and it was revealed that high affinity 5-bp sequences are relatively more enriched in Rok binding regions compared with the core genome. The relative enrichment fold of each 5-bp sequence in the Rok binding regions is positively correlated with the median 8-mer E-score in the PBM experiment, with TACTA to be the most enriched of all 5-bp sequences (Figure 10D). And among all-A/T 5-bp sequences, AAAAA is the least enriched. These genomics features are conserved in other Rok-containing *Bacillus* species, such as *B. amyloliquefaciens* and *B. coagulans*. On the contrary, those *Bacillus* species without Rok, such as *B. anthracis* and *B. thuringiensis*, generally do not display genomic features characteristic for Rok preferred sequences. For example, TACTA, AACTA or ATATA are not rare sequences in *B. anthracis str. Ames* (NC_003997.3, AT-content 64.6%). In this genome, the occurrence frequency of AAAAA is only about 2-fold higher than that of ATATA, and TACTA and AACTA sequences rank 130 and 151 in occurrence among all 512 5-bp sequences, respectively (supplementary dataset S3).

These findings lead us to speculate that the Rok protein and its resident genome may have evolved cooperatively. During evolution, the core genomic genes should have evolved in a way to avoid Rok silencing, resulting in the genome to have a relatively lower AT-content with underrepresentation of AACTA and TACTA sequences, and further promoting the abundance of A-tracts over TpA steps. The correlations between the conserved DNA recognition mechanism of Rok and the characteristic sequence features of Rok resident genomes enable Rok to be an efficient xenogeneic silencer that can distinguish and silence foreign genes selectively.

Up to date, we have studied the DNA recognition mechanisms for all four known families of xenogeneic silencers. Taken all together, a good xenogeneic silencer should has at least two properties. Firstly, it must have the ability to distinguish foreign from self DNA. As different bacteria can have quite different genomic sequence properties and the corresponding foreign DNA sequences are likely to have different features, the DNA binding preferences of the xenogeneic silencers should be correlated with the characteristics of their resident genome. Secondly, it should have a tight enough binding affinity towards target DNA for the suppression of transcription. As the DNA binding affinities for single DNA binding domains of the four xenogeneic silencer families are all relatively weak (*K*_d_ ∼10^−6^ M) at most sequences, oligomerization and cooperative binding (multivalency) is essential for their ability to form stable repressive complexes on DNA.

It is well established that xenogeneic silencers play an important roles in the regulation of bacterial genes related to virulence and drug resistance (51), and Lsr2 was proposed to be a potential target for new antibiotic development (52). The understanding of DNA recognition mechanisms of xenogeneic silencers may provide new antibiotic development strategies by inhibiting the DNA binding ability of xenogeneic silencers.

## DATA AVAILABILITY

The motif-based sequence analysis tools MEME is available at http://meme-suite.org/index.html

The Dali server for Protein Structure Database Searching is available at http://ekhidna2.biocenter.helsinki.fi/dali/oldstyle.html

The Curves+ software for analyzing the structure of nucleic acids is available at https://bisi.ibcp.fr/tools/curves_plus/

The multiple sequence alignment tool Clustal Omega and the Easy Sequencing in PostScript tool ESPript (53) are available at https://www.ebi.ac.uk/Tools/msa/clustalo/ and http://espript.ibcp.fr/ESPript/cgi-bin/ESPript.cgi

Structures of Rok-C^97-191^ and Rok-C^102-185^/Seq1 complex have been deposited at The RCSB Protein Data Bank (www.rcsb.org) under accession numbers 5ZUZ and 5ZUX, respectively.

All chemical shift assignments were deposited at the BioMagResBank (www.bmrb.wisc.edu) under accession number 36187 (Rok-C^97-191^) and 36186 (Rok-C^102-185^/Seq1 complex).

PBM data for Rok and Rok-C^97-191^ (supplementary dataset S1), AT-contents of *Bacillus* genomes and the distribution of Rok homologs (supplementary dataset S2), as well as the occurrence frequencies of 5-bp sequence in *B. subtilis* genome or Rok binding regions (supplementary dataset S3) are presented as supplementary materials. PBM data for H-NS, Lsr2 and MvaT were published previously (14,15).

## Supplementary Material

Supplementary Data
