# Supplementary material for: How bacterial xenogeneic silencer rok distinguishes foreign from self DNA in its resident genome
Source: Nucleic Acids Res. 2018 Sep 25;46(19):10514–29. doi: 10.1093/nar/gky836 (PMC6212790; doi:10.1093/nar/gky836)
Supplement: Supplementary Data [file gky836_supplemental_files.zip › Supplementary Data.docx]

## SUPPLEMENTARY DATA

S1 Dataset. Protein binding microarray data for Rok. (XLSX)

S2 Dataset. AT-contents of *Bacillus* genomes and the distribution of Rok homologs. (XLSX)

S3 Dataset. Occurrence frequencies of each 5-bp sequence in some *Bacillus* genomes and Rok binding regions of *B. subtilis*. (XLSX)
