# Supplementary material for: How bacterial xenogeneic silencer rok distinguishes foreign from self DNA in its resident genome
Source: Nucleic Acids Res. 2018 Sep 25;46(19):10514–29. doi: 10.1093/nar/gky836 (PMC6212790; doi:10.1093/nar/gky836)

Figure S1. Oligomerization states of Rok, Rok-N$^{1-95}$, and Rok-C$^{97-191}$. (A) Elution profile from gel filtration column of purified Rok, Rok-N$^{1-95}$, and Rok-C$^{97-191}$ proteins. The calibration curve was generated using the retention volumes of cytochrome C (12.4 kD), BSA monomer (66.4 kD), and BSA dimer (133 kD). (B) SDS-PAGE analysis of the sample in the gel filtration experiment.

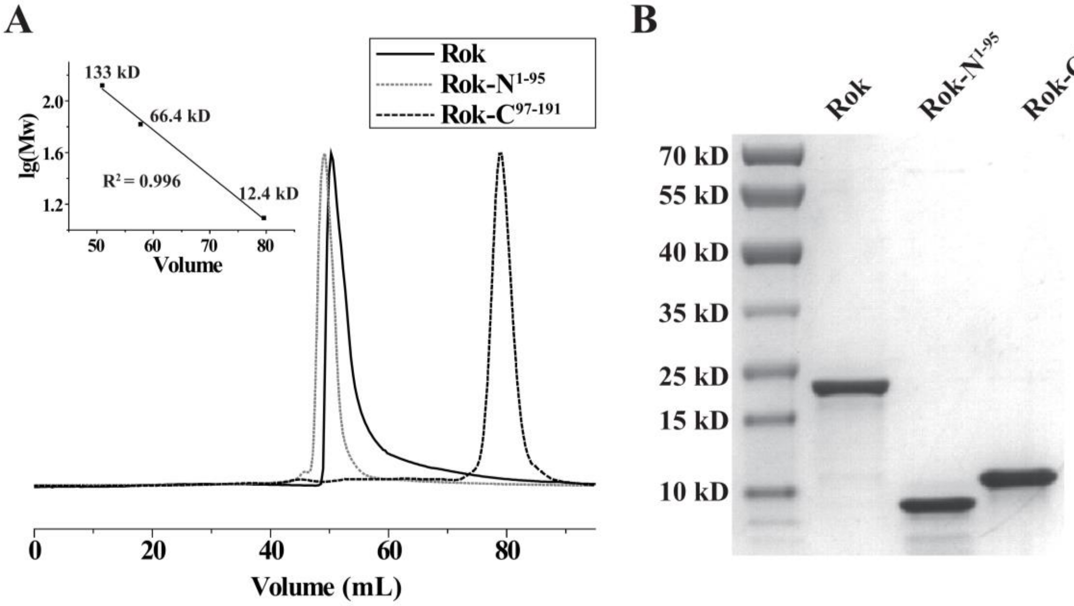

Figure S2 Resonance assignments of the 2D ^{1}H-^{15}N HSQC spectrum of free Rok-C$^{97-191}$.

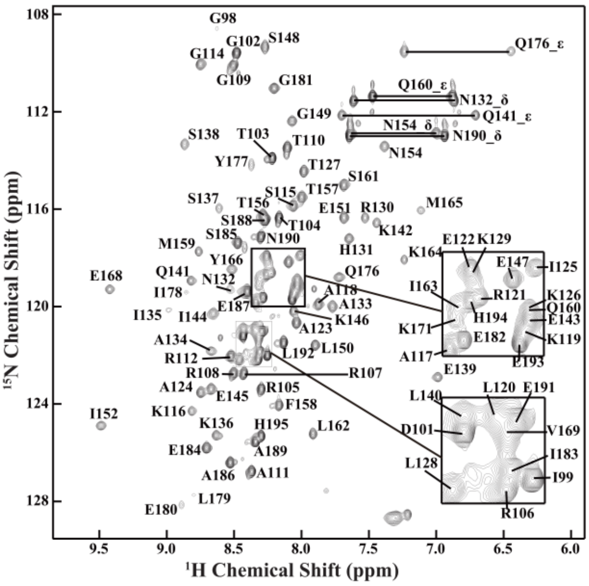

Figure S3. Interaction between d(CGCATATATGCG)$_2$ (3AT) DNA and Rok-C$^{97-191}$ or Rok-C$^{102-185}$. (A) Overlay of the 2D ^{1}H-^{15}N HSQC spectra of free Rok-C$^{97-191}$ (blue) and Rok-C$^{97-191}$/3AT complex (red). (B) 2D ^{1}H-^{15}N HSQC signal intensity ratios between 3AT DNA bound and free Rok-C$^{97-191}$. (C) Combined chemical shifts differences ($\Delta\delta_{comb}$ = $[\delta_{HN}{}^2 + (\delta_N/6.5)^2]^{1/2}$) of the NH signals caused by 3AT DNA. (D) Residues with $\Delta\delta_{comb} > 0.10$ ppm, $\Delta\delta_{comb} > 0.20$ ppm, and $\Delta\delta_{comb} < 0.03$ ppm are represented as red, pink and blue on the protein structure, respectively. (E and F) Overlay of the 2D ^{1}H-^{15}N HSQC spectra of Rok-C$^{97-191}$ and Rok-C$^{102-185}$ in their free (blue) and 3AT DNA bound (red) forms. The molar ratio of protein and DNA is 1:2 for all complex samples.

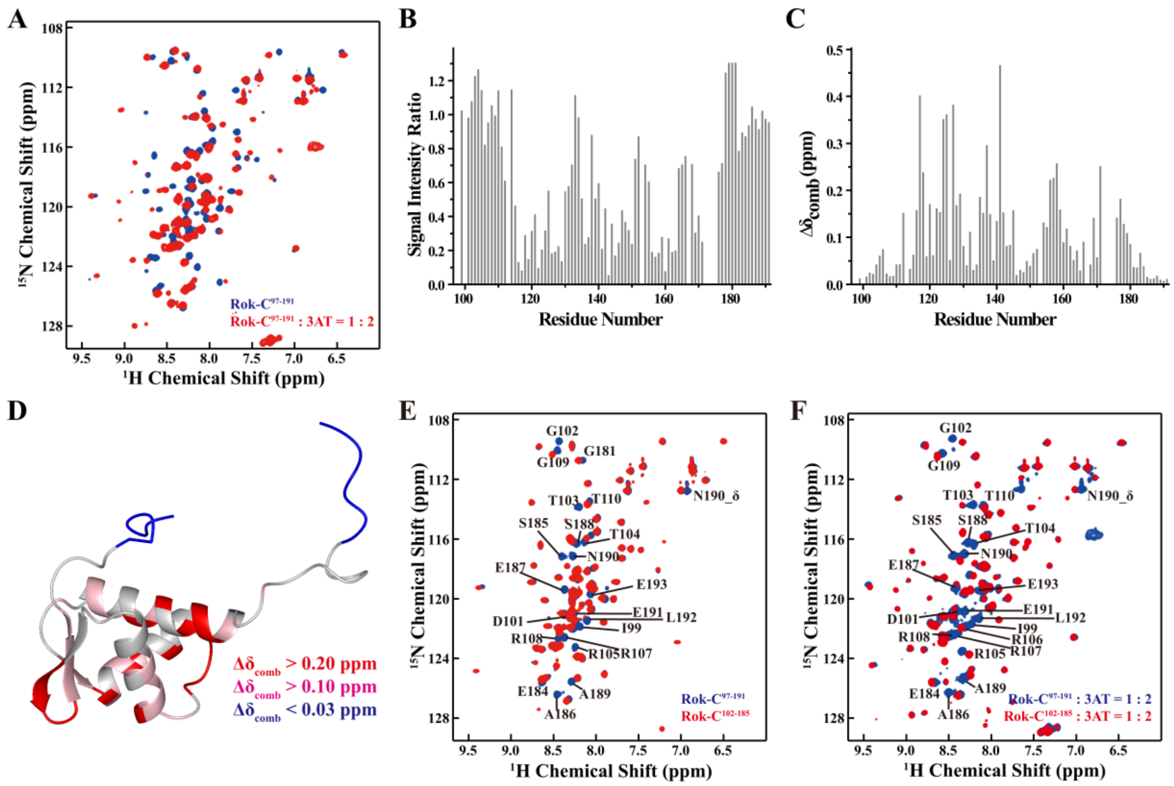

Figure S4. 2D ^{1}H-^{15}N HSQC spectra of Rok-C$^{102-185}$ in the presence of different DNA molecules. The sequence of each DNA is indicated in the figures, and CTAATAACTAGTTATTAG (Seq1) was chosen as the target DNA for the complex structure determination. The molar ratio of protein and DNA is 1:2 for all samples.

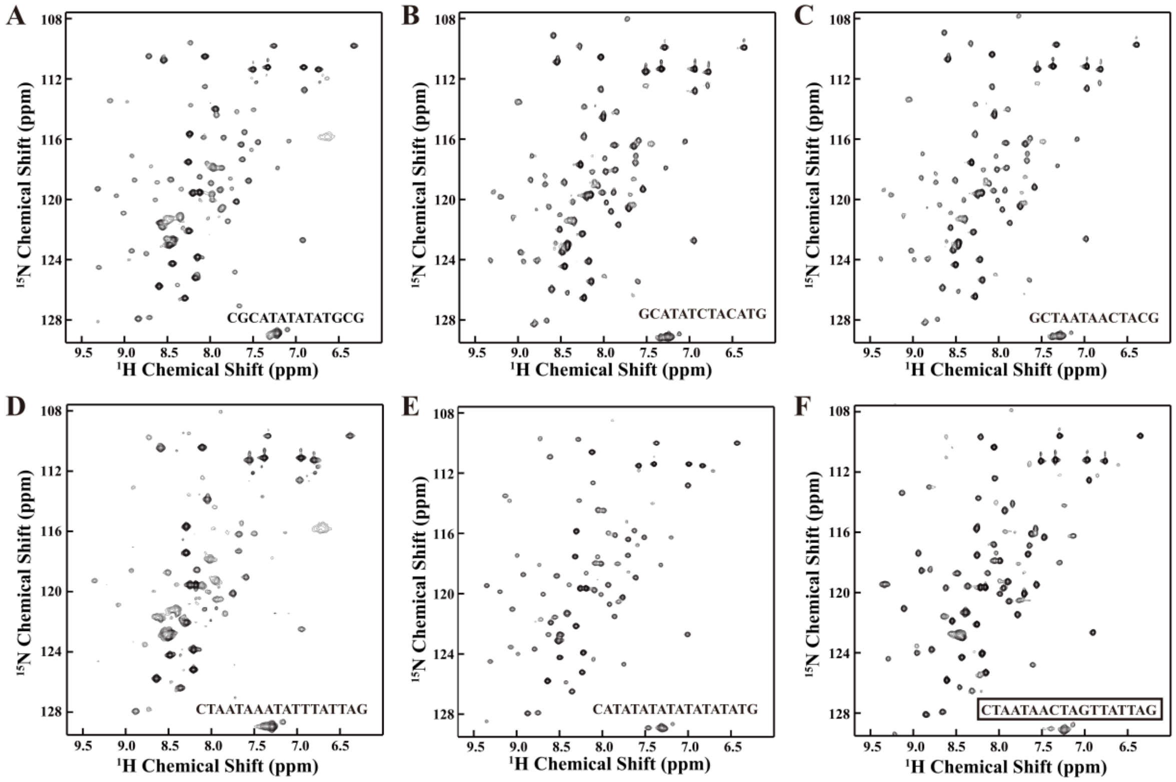

Figure S5. Resonance assignments of Rok-C$^{102\text{-}185}$/Seq1 complex. (A) Assignments of 2D ^{1}H-^{15}N HSQC spectrum of Rok-C$^{102\text{-}185}$/Seq1 complex. (B) Finger print region of 2D ^{1}H-^{1}H NOESY spectra of Seq1 DNA in complex with Rok-C$^{102\text{-}185}$. Intra-residue H1’-H6/H8 NOE peaks are labeled by base type and sequence number (subscript) and sequential connectivity is shown by lines. (C) Assignments of intermolecular NOE signals. N, noise; D, diagonal peaks.

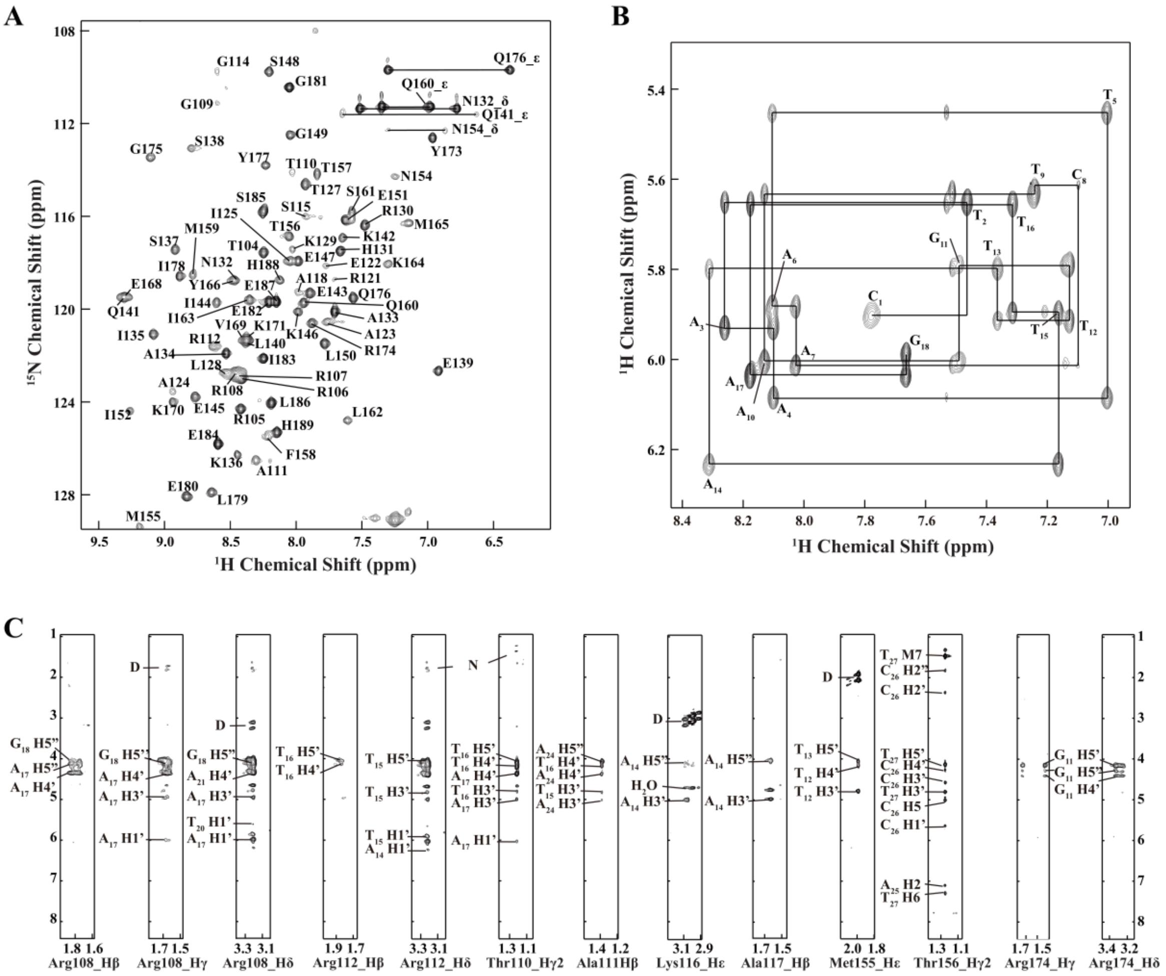

Figure S6. The role of the N-terminal tail in DNA binding. (A and B) Overlay of the 2D ^1H-^{15}N HSQC spectra of Rok-$C^{102\text{-}185}$ and Rok-$C^{114\text{-}185}$ in their free (A) and Seq1 DNA bound forms (B). (C) Isothermal titration curves of Seq1 DNA with Rok-$C^{102\text{-}185}$ and Rok-$C^{114\text{-}185}$.

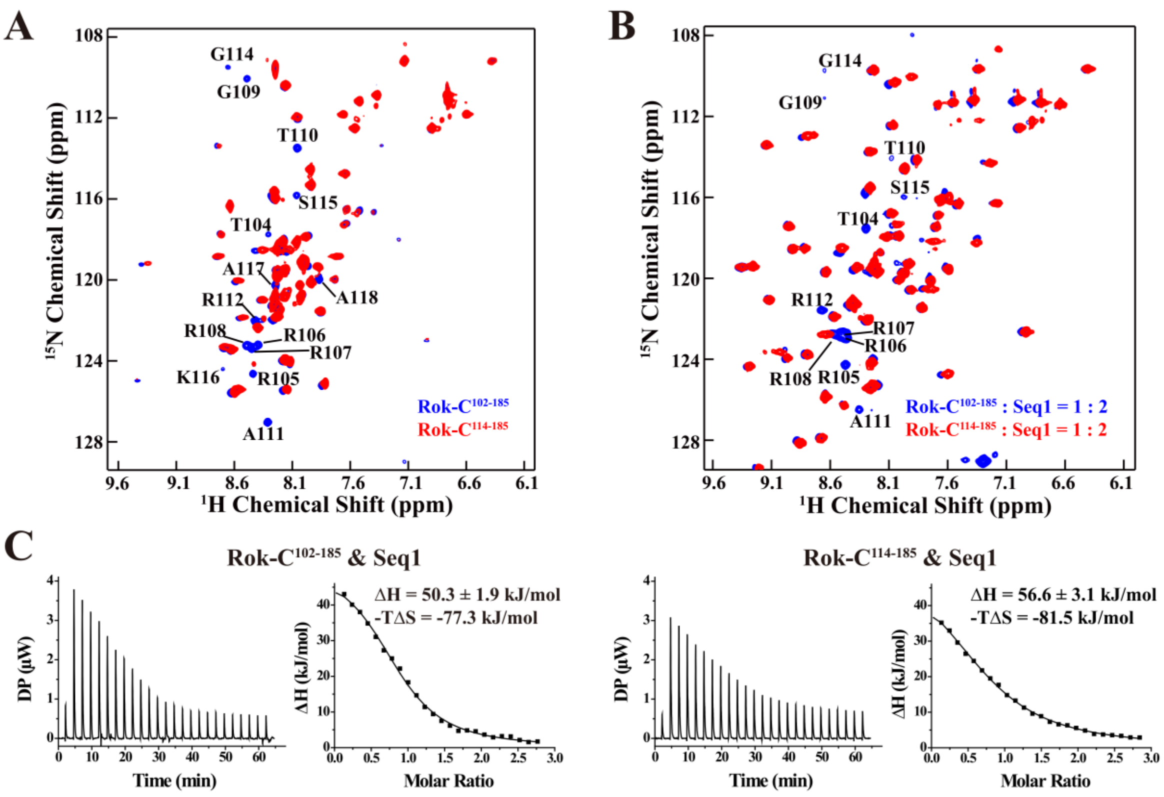

Figure S7. The influence of DNA minor groove binder netropsin and major groove binder methyl green on the binding of Rok-C$^{114\text{-}185}$ with Seq1 DNA. (A) 2D ^{1}H-^{15}N HSQC spectra of Rok-C$^{114\text{-}185}$ with different ratios of netropsin and Seq1 DNA. The molar ratios of netropsin, Seq1 DNA, and Rok-C$^{114\text{-}185}$ are 0:2:1 (blue), 4:2:1 (yellow), 8:2:1 (green), 16:2:1 (red), and 0:0:1 (black). (B and C) The impact of methyl green on the 2D ^{1}H-^{15}N HSQC spectra of Rok-C$^{114\text{-}185}$ with 2-fold Seq1 DNA (B) or free Rok-C$^{114\text{-}185}$ (C). Precipitates were observed when methyl green was added to the samples, leading to significant signal intensity attenuation. The molar ratios of methyl green and Rok-C$^{114\text{-}185}$ are 0:1 (black), 4:1 (red), and 8:1 (blue).

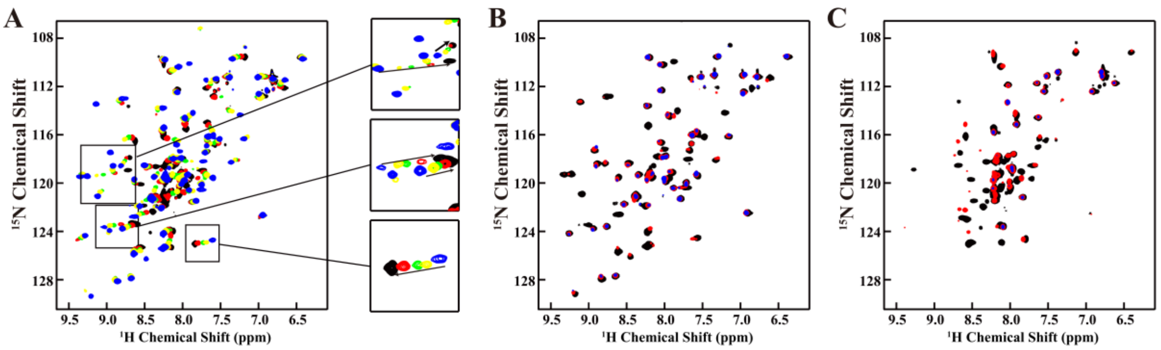

Figure S8. Comformational changes of Rok-C$^{102\text{-}185}$ upon DNA binding. (A)The mean structures of free (hot pink) and Seq1-DNA-bound (cyan) Rok-C$^{102\text{-}185}$ are aligned according to the β-sheet. (B, C, and D) Overlay of the 2D ^{1}H-^{15}N HSQC spectra (C) and the methyl regions of the 2D ^{1}H-^{13}C HSQC spectra (D) of free (hot pink) and Seq1-DNA-bound (cyan) Rok-C$^{102\text{-}185}$. Residues with combined NH chemical shifts differences ($\Delta\delta_{comb}$) larger than 0.10/0.20 ppm are represented as pink/red on the complex structure (B).

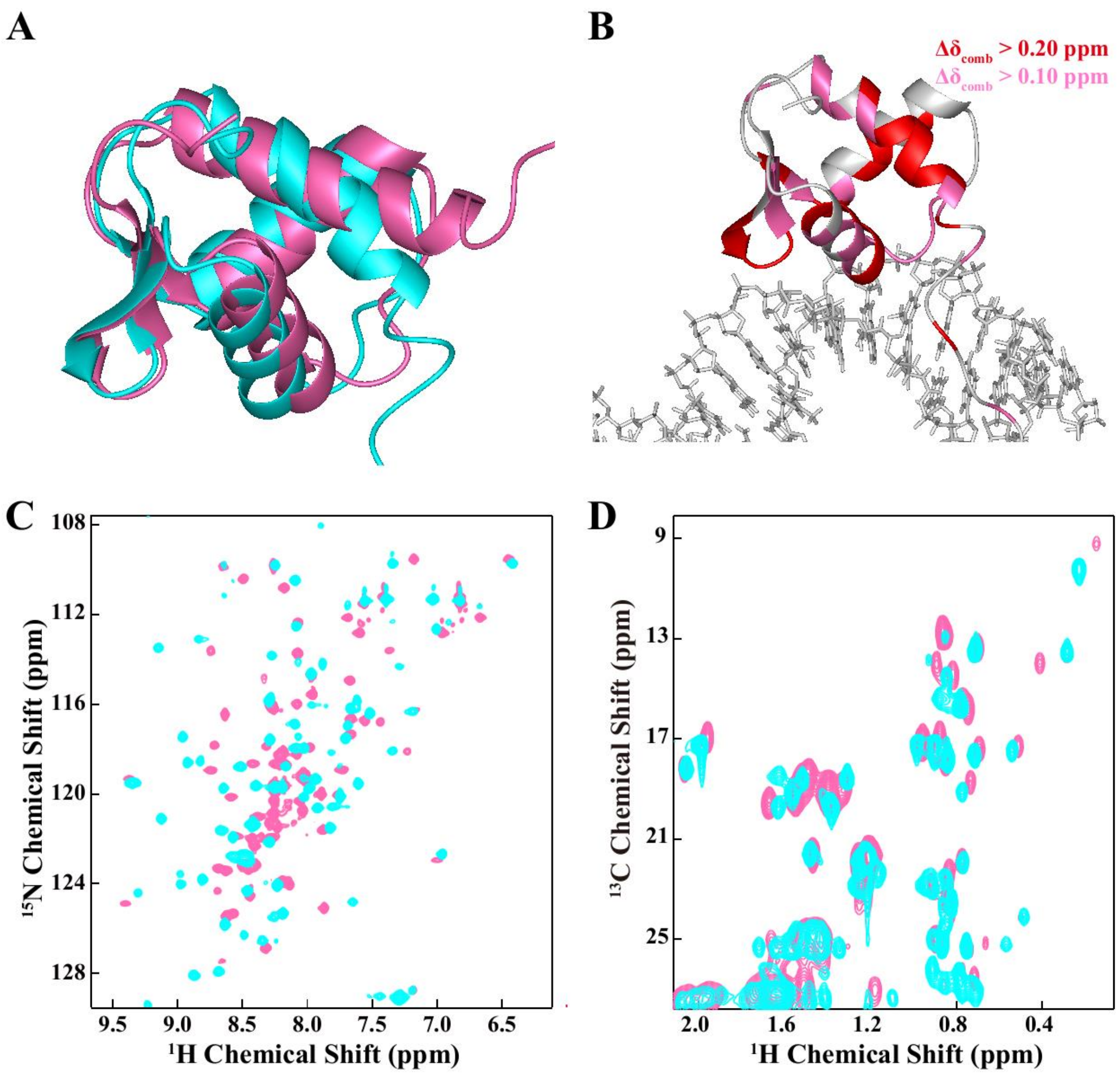

Figure S9. 2D ^{1}H-^{15}N HSQC spectra of N154A (A), N154S (B), and N154Q (C) mutants of Rok-C$^{114-185}$, and the hydrogen bond between the NH group of T157 and the sidechain C=O group of N154 (D).

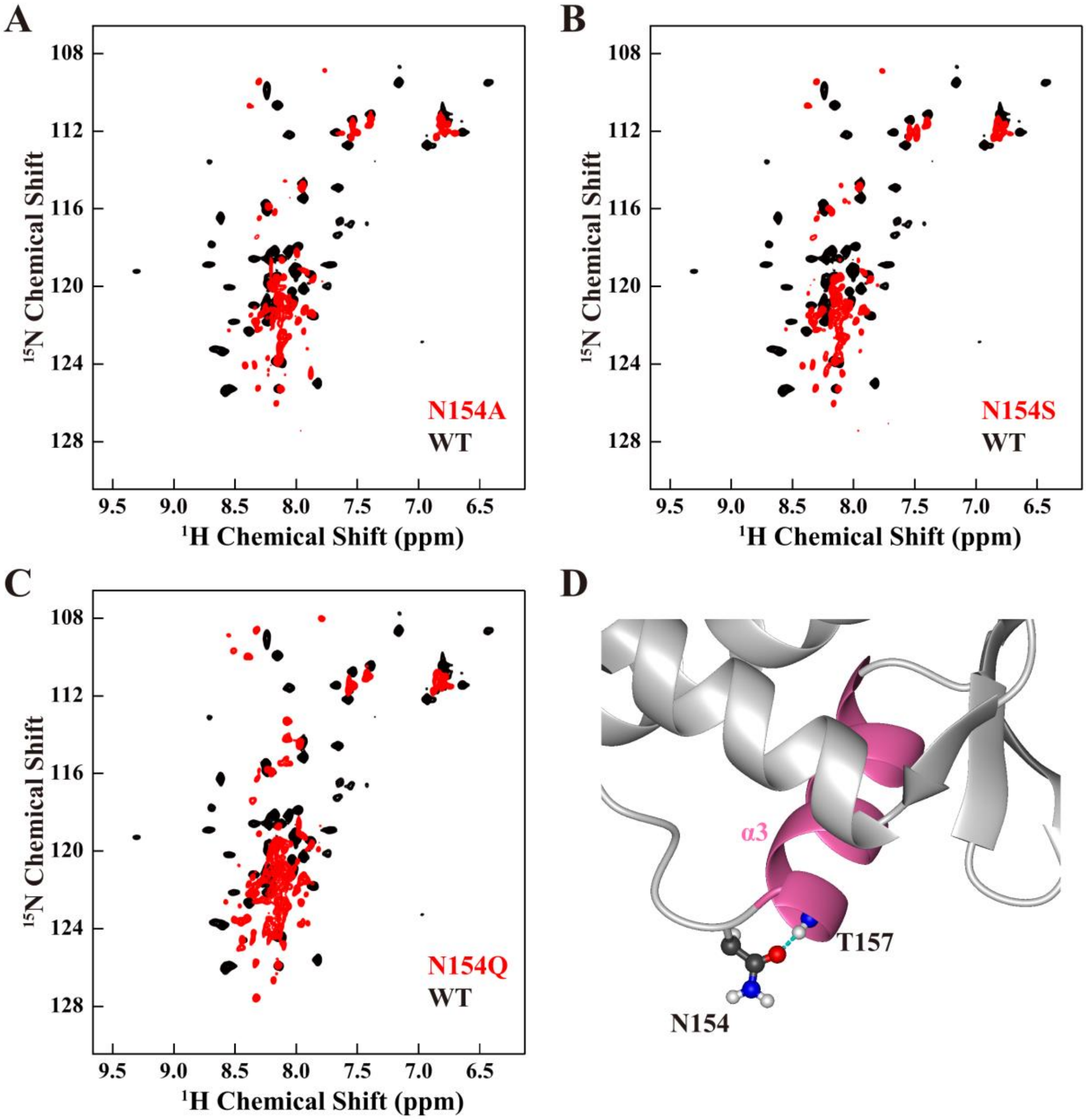

Figure S10. Chemical shift perturbations of Seq1 DNA on WT Rok-C$^{114\text{-}185}$ and its mutants R174A, T156A, T157A, N154D, K171A, K116A, K136A, and K164A. 2D ^{1}H-^{15}N HSQC spectra of free and Seq1-DNA-bound WT and mutant Rok-C$^{114\text{-}185}$ are overlaid together. For the Seq1-DNA-bound form, the molar ratio of Seq1 DNA and protein are 2:1. The combined NH chemical shift differences upon Seq1 DNA binding of the mutant (blue) and WT (red) proteins are calculated and compared.

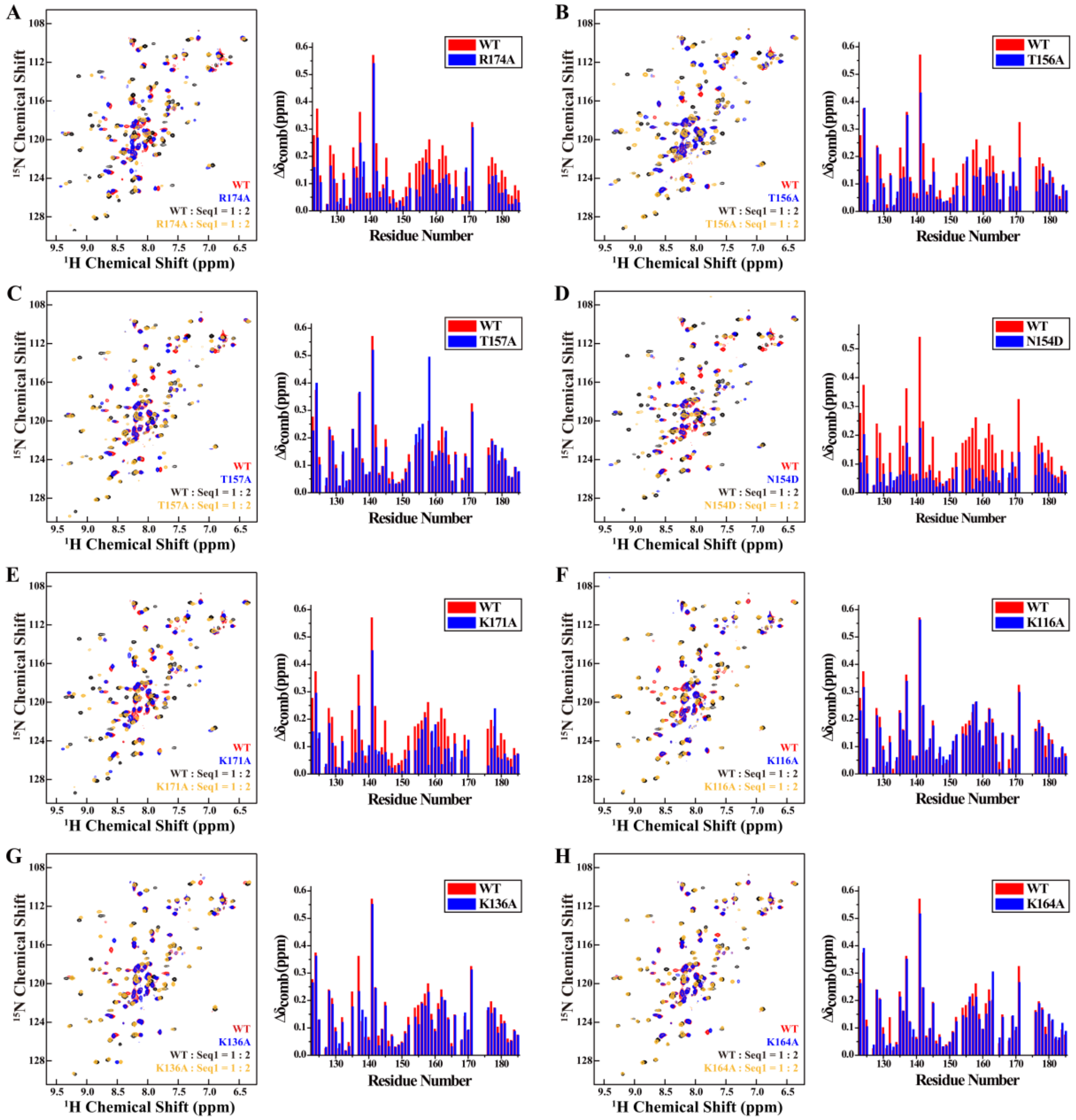

Figure S11. ITC curves for R174A, T156A, T157A, N154D, K171A, K116A, K136A, and K164A mutants of Rok-C$^{114\text{-}185}$ with Seq1 DNA. N/D, not detectable.

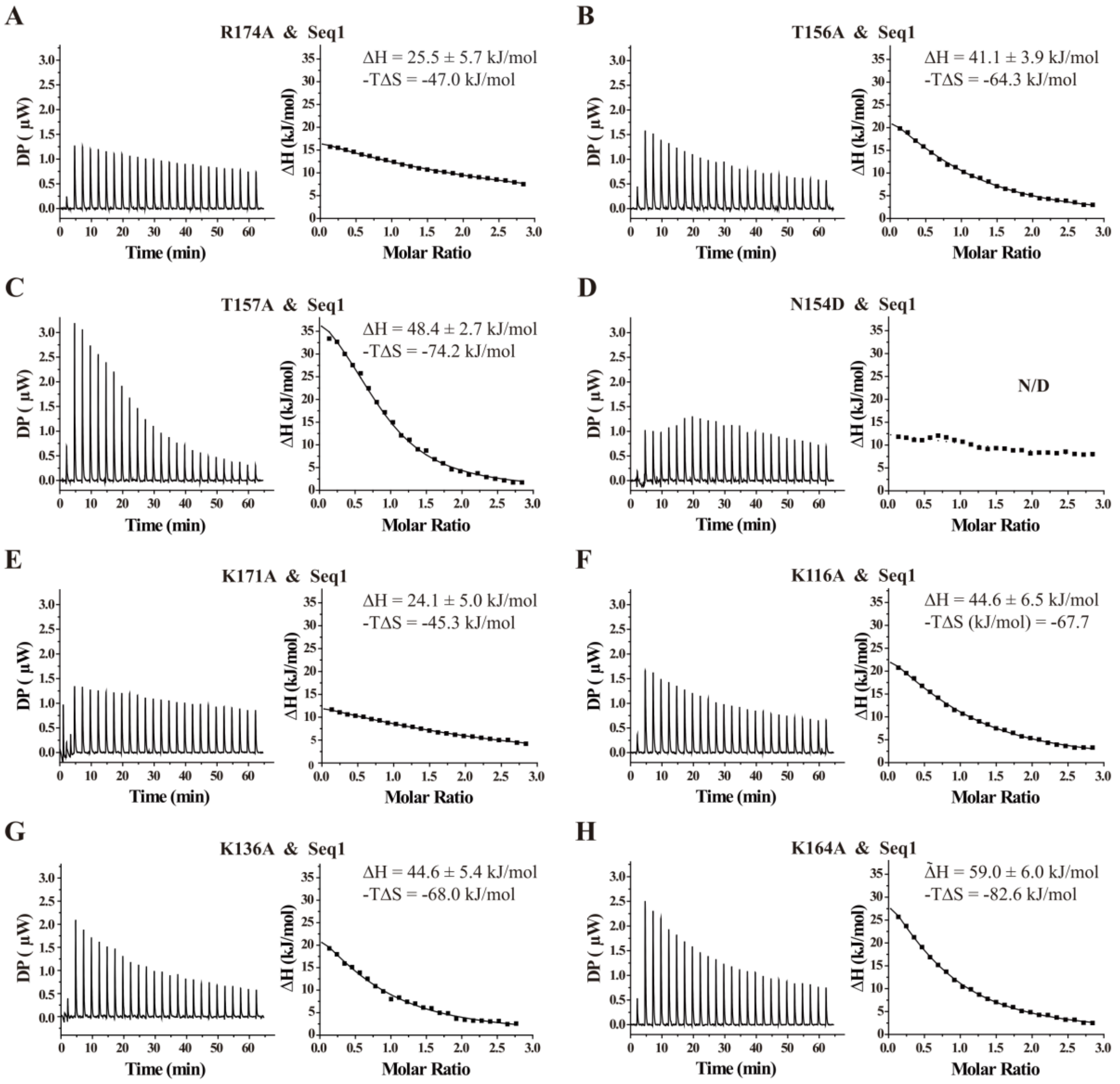

Figure S12. Comparison of the binding of Rok-C$^{102\text{-}185}$ to Seq1 and 8AT DNA. (A) 2D ^{1}H-^{15}N HSQC spectra of Rok-C$^{102\text{-}185}$/8AT complex (red) and Rok-C$^{102\text{-}185}$/Seq1 complex (black). The molar ration of protein and DNA are 1:2. (B) Combined chemical shift differences of NH signals of Rok-C$^{102\text{-}185}$ bound by 8AT and Seq1 DNA. (C) Residues with $\Delta\delta_{comb}$ > 0.10/0.05 ppm are colored red/pink on the structure of Rok-C$^{102\text{-}185}$/Seq1 complex. Base pairs which are different between Seq1 and 8AT are colored blue.

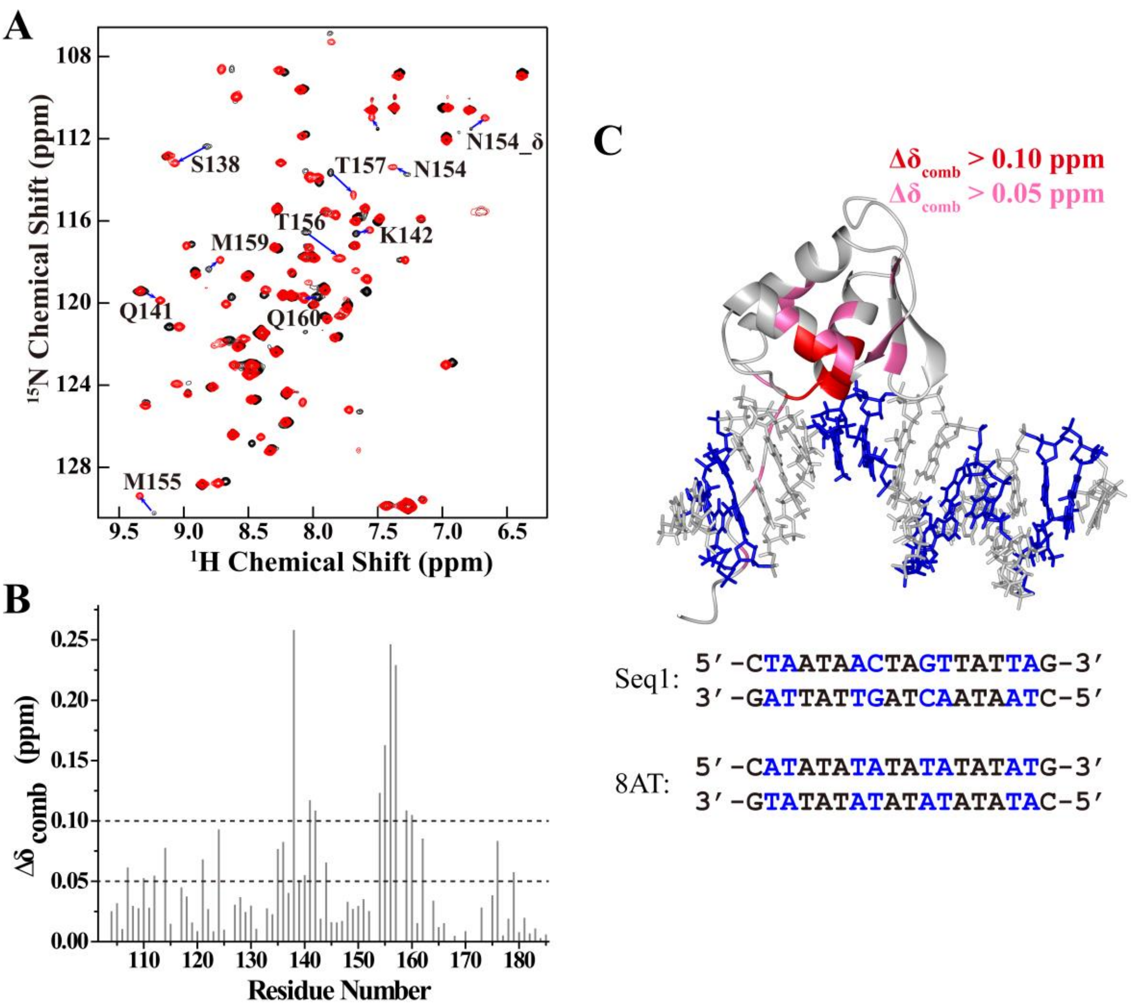

Figure S13. Distribution of Rok homologs. The numbers of Rok homologs found in different bacterial species are indicated, revealed from a Blastp search against the non-redundant database of NCBI with the sequence of Rok-$C^{114\text{-}185}$. (*106 *Bacillus* species are omitted, which contain totally 133 Rok homologs).

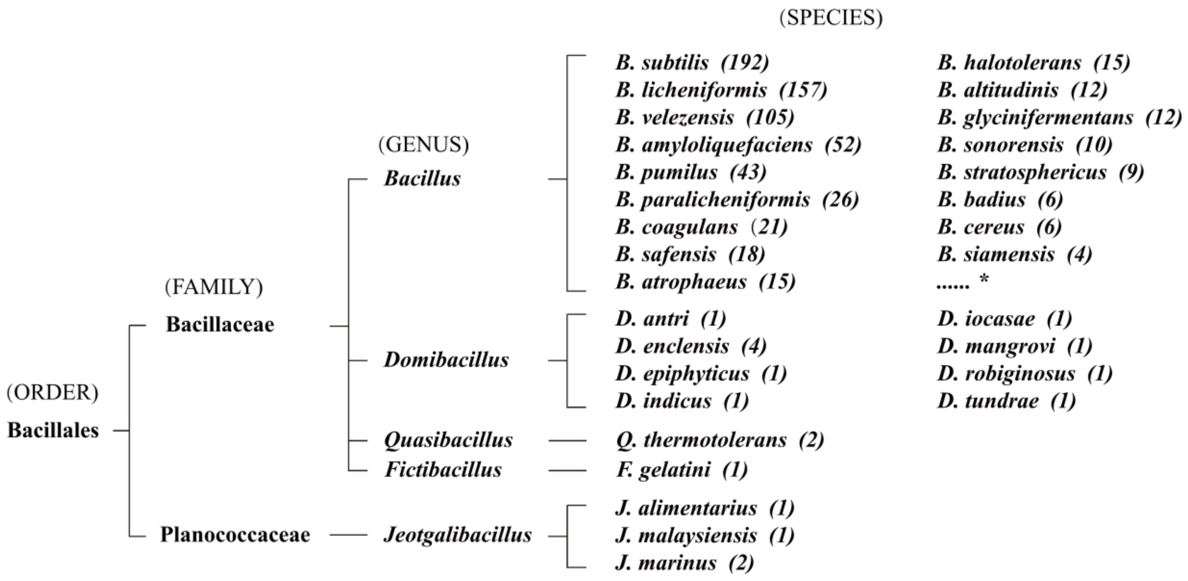

Supplement: Supplementary Data [file gky836_supplemental_files.zip › Supplementary Figures.pdf]
